# Surface and Interface Engineering for Nanocellulosic Advanced Materials

**DOI:** 10.1002/adma.202002264

**Published:** 2020-09-09

**Authors:** Xianpeng Yang, Subir Kumar Biswas, Jingquan Han, Supachok Tanpichai, Mei‐Chun Li, Chuchu Chen, Sailing Zhu, Atanu Kumar Das, Hiroyuki Yano

**Affiliations:** ^1^ Laboratory of Active Bio‐Based Materials Research Institute for Sustainable Humanosphere (RISH) Kyoto University Uji Kyoto 611‐0011 Japan; ^2^ College of Materials science and Engineering Nanjing Forestry University Nanjing 210037 P. R. China; ^3^ Learning Institute King Mongkut's University of Technology Thonburi Bangkok 10140 Thailand; ^4^ Department of Forest Biomaterials and Technology Swedish University of Agricultural Sciences Umeå SE‐90183 Sweden

**Keywords:** cellulose nanocrystals, cellulose nanofibers, nanocelluloses, reinforcement, surface modification

## Abstract

How do trees support their upright massive bodies? The support comes from the incredibly strong and stiff, and highly crystalline nanoscale fibrils of extended cellulose chains, called cellulose nanofibers. Cellulose nanofibers and their crystalline parts—cellulose nanocrystals, collectively nanocelluloses, are therefore the recent hot materials to incorporate in man‐made sustainable, environmentally sound, and mechanically strong materials. Nanocelluloses are generally obtained through a top‐down process, during or after which the original surface chemistry and interface interactions can be dramatically changed. Therefore, surface and interface engineering are extremely important when nanocellulosic materials with a bottom‐up process are fabricated. Herein, the main focus is on promising chemical modification and nonmodification approaches, aiming to prospect this hot topic from novel aspects, including nanocellulose‐, chemistry‐, and process‐oriented surface and interface engineering for advanced nanocellulosic materials. The reinforcement of nanocelluloses in some functional materials, such as structural materials, films, filaments, aerogels, and foams, is discussed, relating to tailored surface and/or interface engineering. Although some of the nanocellulosic products have already reached the industrial arena, it is hoped that more and more nanocellulose‐based products will become available in everyday life in the next few years.

## Graphical abstract

Nanocelluloses, the natural reinforcement for plants, are incredibly strong and stiff, and are therefore under great scrutiny for incorporation in sustainable, ecofriendly, and strong man‐made materials. Nanocelluloses enable dramatic tailoring of surface chemistry, interface interactions, and functionalities via chemical and/or physical surface and interface engineering that permit assembly of diverse nanocellulosic materials. Some of the promising progress in this area is reviewed.

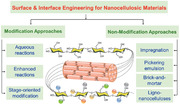

## 1 Introduction

**Figure 1:**
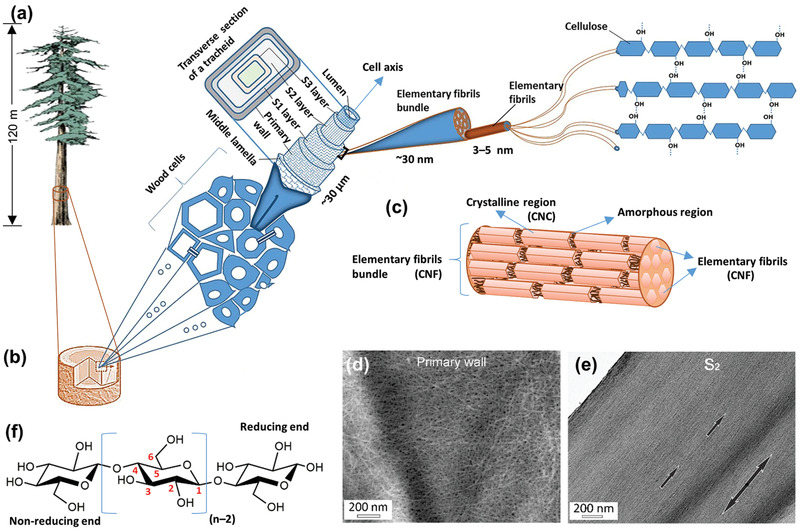
The skeleton of trees reinforced by nanoscale cellulose fibrils. a) Schematic of a coast‐redwood tree (*Sequoia sempervirens*) found in California that may reach >120 m in height. Adapted under the terms of the CC‐BY Creative Commons Attribution 4.0 International license (https://creativecommons.org/license/by/4.0).^[^ ^24^ ^]^ Copyright 2017, The Authors, published by Springer Nature. b) The huge body of a tree is mechanically supported by the wood, which is a nanocomposite of hierarchical cellulose fibrils (reinforcing phase) embedded in the hemicelluloses and lignin (matrix phase). Adapted under the terms of the CC‐BY Creative Commons Attribution 4.0 International license (https://creativecommons.org/license/by/4.0).^[^ ^5^ ^]^ Copyright 2019, The Authors, published by Springer Nature. c) Schematic of a cellulose elementary fibril or microfibril bundle showing the organization of crystalline and amorphous regions in the elementary fibrils. After extraction, the elementary fibrils and their bundles are called cellulose nanofibers (CNFs), and the crystalline regions are called cellulose nanocrystals (CNCs). d) Scanning electron microscopy (SEM) image of elementary fibril bundles directly seen in the primary wall of a wood cell after pulping. e) Transmission electron microscopy (TEM) image of the characteristic orientation of the elementary fibril bundles directly seen in the S_2_ secondary cell wall layer. The single‐headed arrows indicate the elementary fibril orientations and the double‐headed arrow indicates the longitudinal cell axis (growth axis). Adapted with permission.^[^ ^6^ ^]^ Copyright 2014, Springer Nature. f) Basic chemical structure of cellulose with the numbering system of carbon atoms in the anhydroglucose unit; *n* ≈ 2000 to 27 000, depending on the cellulose source material.^[^ ^22^ ^]^

Trees grow upright and tall in response to positive phototropism and negative geotropism.^[^ ^1^ ^]^ Some tree species may grow beyond 120 m in height (**Figure** **1**a). The fall of a 113 m tall coast‐redwood tree, named the Dyerville Giant, in 1991 in Humboldt Redwoods State Park in California, was so severe that its fall was read on a nearby seismograph.^[^ ^2^ ^]^ How do trees support their upright massive bodies? The support originates from the hierarchically designed nanocomposite traditionally known as wood, the building blocks (cells) of which are reinforced by incredibly strong and stiff nanoscale fibrils of extended cellulose chains, which are known as cellulose elementary fibrils or microfibrils (3–5 nm wide) and the bundles thereof (15–50 nm wide) (Figure 1b–f).^[^ ^3^, ^4^, ^5^, ^6^, ^7^ ^]^ These fibrils are highly crystalline with a tensile strength (σ) of 2–7.7 GPa, a crystal elastic modulus (*E*) of ≈140 GPa, and a density (ρ) of ≈1.6 g cm^−3^.^[^ ^8^ ^]^ To place their properties into perspective, they are mechanically comparable to Kevlar, approximately seven times stronger but five times lighter than steel, and their thermal expansion is low (see **Table** **1**). These marvelous natural nanomaterials are ubiquitous and are found in all woody and non‐woody plants and other sources, such as bacteria, algae, and sea‐animal tunicates.^[^ ^7^, ^9^, ^10^, ^11^ ^]^ Furthermore, they are readily extractable, biodegradable, renewable, and carbon neutral. Therefore, nanoscale cellulose fibrils have received extensive attention in the early 21st century to fabricate ecofriendly, lightweight, and robust (composite) materials, and related topics may constitute the most mature studies of wood nanotechnology.

**Table 1:** Properties of nanocellulose compared to various other reinforcing materials. Adapted with permission.^[^ ^7^ ^]^ Copyright 2011, Royal Society of Chemistry

Material	ρ [g cm^−3^]	σ [GPa]	*E* [GPa]	CTE^a)^ [ppm K^−1^]	Refs.
Kevlar‐49 fiber	1.4	3.6–4.1	124–131	–2.0	^[^ ^25^, ^26^ ^]^
Carbon fiber	1.8	1.5–5.5	150–500	–0.6	^[^ ^25^ ^]^
Mild steel	–	0.4–0.6	194–243	–	^[^ ^27^ ^]^
High strength steel	–	0.8–0.9	207–242	–	^[^ ^27^ ^]^
Stainless steel	7.8	0.4–1.8	193–204	10.2–17.2	^[^ ^25^ ^]^
Clay nanoplatelets	–	–	170	–	^[^ ^28^ ^]^
Carbon nanotubes	–	11–63	270–950	–	^[^ ^29^ ^]^
Nanochitin	1.6	1.6–3	41–70^b)^	21	^[^ ^30^ ^]^
Nanocellulose	1.6	2–7.7	≈140^b)^	0.1^c)^, 6^d)^	^[^ ^8^, ^31^ ^]^

a) CTE, coefficient of thermal expansion

b) *E* of the crystalline region

c) CTE of the all‐cellulose composite measured using a thermomechanical analyzer

d) CTE of the crystalline region measured by X‐ray diffraction.

Nanoscale cellulose fibrils that are extracted irrespective of cellulosic sources have two generic forms.^[^ ^7^, ^11^, ^12^ ^]^ The first fibril form is semicrystalline, typically 3–50 nm wide and ≈1–3 µm long, with a high aspect ratio and flexibility and is termed cellulose nanofibers or nanofibrils (CNFs). Typically, CNFs are produced from lignocellulosic materials (i.e., wood and plants—in particulate forms). The extraction process starts with the chemical purification to liberate the cellulose fibers (also called pulp fibers) from the matrix of lignin and hemicelluloses.^[^ ^13^ ^]^ The process is well known in the pulp and paper industries as the Kraft/sulfate pulping and sulfite pulping; the most common laboratory purification process is called “Wise method,” where repeated treatment of the lignocellulosic particles with acidified sodium chlorite (NaClO_2_) and potassium hydroxide (KOH) is done to remove the matrices.^[^ ^14^ ^]^ The purified cellulose fibers then can be directly nanofibrillated by mechanical treatment in a never‐dried condition either using grinder, high‐pressure homogenizer/microfluidizer, or high intensity ultrasonics.^[^ ^13^ ^]^ To facilitate nanofibrillation of the purified cellulose materials, a chemical or enzymatic pretreatment can be applied before the mechanical treatments.^[^ ^13^ ^]^ Those pretreatments help swell the fiber wall that effectively loosen the interfibrillar hydrogen bonds and hence facilitate the extraction of the finer fibrils during the subsequent mechanical treatments. The enzymatic treatment does not modify the cellulose because it only attacks the amorphous regions of the cellulose microfibrils, and thus reduces the energy requirements during the mechanical fibrillation by a homogenizer/microfluidizer.^[^ ^13^, ^15^ ^]^ A chemical treatment, e.g., TEMPO (2,2,6,6‐tetramethylpiperidine‐1‐oxyl radical)‐mediated oxidation^[^ ^4^, ^13^, ^16^ ^]^ and carboxymethylation,^[^ ^13^, ^17^ ^]^ usually introduces charges to the cellulose fibrils (i.e., modifies its surface), which helps the fiber to swell more by electrostatic repulsion thereby facilitates nanofibrillation. For example, TEMPO‐mediated oxidation can introduce carboxylate charges (so does the carboxymethylation) to the cellulose by oxidation of the hydroxyl groups. After TEMPO pretreatment of the cellulose fibers followed by disintegration via a mild magnetic stirring, the CNFs can be easily obtained with a native elementary width of 3–5 nm. Therefore, the TEMPO‐oxidized CNFs, typically abbreviated as TOCNs, recently received an enormous attention.

The second fibril form is highly crystalline, short (typically <500 nm) and thin (typically 3 nm to ≈20 nm wide), with a low‐aspect‐ratio and rigid fibrils obtained by hydrolyzing amorphous parts of the elementary fibrils (see Figure 1c), and is known as cellulose nanowhiskers or nanorods or nanocrystals (CNCs). Concentrated sulfuric acid (H_2_SO_4_) hydrolysis is ubiquitously used, which can directly produce CNCs from the purified pulp as well as from the CNFs.^[^ ^18^ ^]^ The CNFs and CNCs both forms are identified collectively as “nanocelluloses.”

Nanocelluloses possess abundant hydroxyl groups (—OH) on their surface from the cellulose (Figure 1f) and hemicellulose molecules (a hemicellulose coating may present on the plant‐derived nanocellulose surface, and the amount may vary with the source and extraction method, and may be as high as 30%).^[^ ^19^ ^]^ The hydroxyl groups are important to form strong intricate internanofibrillar networks and/or nanocellulose (hydrophilic) matrix interactions via hydrogen bonding to promote the material performance. However, the hydroxyl groups hinder the transfer of the remarkable mechanical and physical nanocellulose material properties by posing difficulties in cellulose nanofibrillation, a good dispersion in hydrophobic media and matrices (severe at high share), and good nanocellulose–(hydrophobic)matrix interactions for interfacial stress transfer, along with a susceptibility to moisture. For this reason, various chemical and nonchemical surface and interface engineering strategies (e.g., targeted modification of reactive hydroxyl groups, use of coupling agents and amphiphilic molecules, and biomimetic and layer‐by‐layer assemblies) have been explored. Some of these strategies, which enhance the mechanics, can add novel functionalities to yield advanced nanocellulosic materials that are suited for structural, (opto)electronic, photonic, and medical applications, among many others.

We review the recent progress of surface and interface engineering strategies to fabricate nanocellulose‐based advanced materials. Related topics, which are core to nanocellulose‐based materials research, have been discussed and reviewed extensively in recent literature.^[^ ^7^, ^10^, ^11^, ^12^, ^20^, ^21^, ^22^, ^23^ ^]^ Here, we focus mainly on surface and interface engineering strategies for nanocellulosic materials in which the reinforcement that is provided by nanocelluloses (like in their original environment) is important. Thick structural materials, 3D printing ink, films, filaments/fibers, hydrogels, aerogels, and foams are covered because their industrial application is highly likely. The highlight of this review is the information on the state‐of‐the‐art interface engineering strategies that enables nanocellulosic materials processing in aqueous media and/or without nanocellulose chemical modification for maximized performance and environmental soundness.

## 2 General Modification of Nanocelluloses

**Figure 2:**
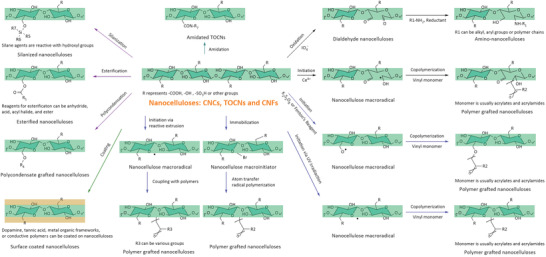
The main surface chemistries of nanocelluloses discussed in Sections 3, 4, 5. Note that some important reactions, such as etherification, click chemistry, etc., are not covered since we focus more on engineering aspects rather than chemistry. We refer to some excellent reviews for more details on the chemistries of nanocelluloses.^[^ ^33^, ^34^, ^38^, ^39^ ^]^

The reactivity of nanocelluloses, which are mainly composed of cellulose, is retained, and chemical modification of nanocelluloses is an issue more than chemistry.^[^ ^32^, ^33^ ^]^ First, nanocelluloses tend to be produced as a dilute aqueous suspension. In many cases, chemical modification, such as esterification and silylation, occurs on the nanocellulose hydroxyl groups.^[^ ^33^, ^34^ ^]^ These reactions are sensitive to water and are performed in polar organic solvents. Pretreatment and posttreatment by solvent exchange are tedious but inevitable, which makes the modification process environmentally unfriendly and difficult to scale up.^[^ ^35^ ^]^ Second, because of their high viscosity, the modification is typically present at low concentrations. The reaction efficiency is low and the reagent consumption increased. Third, the nanocellulose crystallinity and/or morphology may change under severe reaction conditions. Fourth, different nanocelluloses, CNCs, TOCNs, and CNFs, have different features, which require different modification processes. CNCs are spindle‐like crystals with or without charged surface groups, and they depend on the isolation method.^[^ ^36^ ^]^ TOCNs are individual nanofibers with carboxyl surface groups.^[^ ^37^ ^]^ CNFs are isolated mainly from wood pulp by mechanical disintegration, and CNFs branches tend to form intricate networks.^[^ ^37^ ^]^ While CNCs and TOCNs comprise almost 100% cellulose, the CNFs contain a hemicellulose coating, the content of which varies with the source and extraction method, and may be as high as 30%.^[^ ^19^ ^]^ Because of the reasons above, it remains challenging to modify the nanocellulose surface simply and efficiently. Several excellent reviews have been published on nanocellulose chemical modification and they focus on the nanocellulose reaction type.^[^ ^33^, ^34^, ^38^, ^39^ ^]^ Herein, we focus on chemical modification of nanocelluloses from different aspects. In Section 3, we summarize recent developments of aqueous reactions of nanocelluloses. Water replacement by organic solvents can be omitted if water is used as a reaction medium. In Section 4, we focus on the surface modification of nanocelluloses with unconventional and enhanced processes, such as solvent‐free reactions. In Section 5, we discuss the modification stage. CNCs, TOCNs, and CNFs that are isolated by a “top‐down” approach, are typically modified after isolation. The modification can be conducted before and during nanocellulose isolation, during composite processing, and after material formation. It should be noted that matrix tailoring is also an important approach to improve the interface compatibility, which is not discussed here. The main surface chemistries of nanocelluloses mentioned in Sections 3, 4, 5 are illustrated in **Figure** **2**.

## 3 Aqueous Modification of Nanocelluloses

As mentioned previously, the chemical modification of nanocellulose in water is of great interest, particularly in terms of hydrophobic modification. In this section, we focus on recent developments in the aqueous modification of nanocelluloses, including nanocellulose coupling, grafting from nanocelluloses, and nanocellulose surface coating.

### 3.1 Coupling of Nanocelluloses in Aqueous Conditions

Nanocellulose coupling means that molecules are linked on the nanocellulose surfaces, including small molecules and polymers. Physical adsorption on nanocelluloses in water through noncovalent interactions, such as ionic–electrostatic interactions, hydrogen bonds, hydrophilic affinity, and van der Walls forces, is described in previous reviews.^[^ ^33^, ^34^ ^]^ Amidation between TOCNs and amines, and nanocellulose click chemistry have also been summarized previously.^[^ ^34^ ^]^ In this section, we focus mainly on the recently reported covalent coupling of nanocellulose in water.

Nanocellulose oxidation by periodate is an aqueous reaction, which has been used to induce functional groups for further hydrophobic modification, that is, a periodate oxidation/reductive amination strategy.^[^ ^40^, ^41^, ^42^, ^43^, ^44^, ^45^, ^46^ ^]^ In 2014, Guigo et al. oxidized CNFs with NaIO_4_ and then conducted reductive amination with benzylamine.^[^ ^40^ ^]^ Oxidation occurred initially on the surface, and the resultant carbonyl moieties were recombined into hemiacetals.^[^ ^40^ ^]^ During the modification process, solvent‐exchange and organic solvents were avoided and the reaction occurred at room temperature, whereas purification was achieved by repeated centrifugation at 12 000 g.^[^ ^40^ ^]^ Interestingly, the CNF surface was made hydrophobic with benzylamide through reductive amination, which was also performed in water.^[^ ^40^ ^]^ Although a large excess of benzylamide was used, only half of the hemiacetals could be converted owing to steric reasons.^[^ ^40^ ^]^ Later, a water‐soluble polymer, Jeffamine polyetheramine M2070, was grafted onto oxidized CNCs via the periodate oxidation/reductive amination strategy.^[^ ^41^ ^]^ At a low degree of oxidation, the oxidation occurs exclusively at the surface of nanocelluloses, which keeps the crystallinity and microfibrillar integrity intact. However, the crystallinity and structure of nanocelluloses may deteriorate, because the oxidation can also progress toward the core of nanocelluloses with a high degree oxidation.^[^ ^40^, ^41^ ^]^ Alkyl amines with different chain lengths were attached to oxidized CNCs to adjust their hydrophobicity.^[^ ^42^, ^45^ ^]^ After amination, highly transparent CNC suspensions were obtained due to finely nanosized structures.^[^ ^45^ ^]^ The periodate oxidation/reductive amination strategy is promising because of the aqueous medium, mild conditions, and wide versatility to different nanocelluloses. Further, this strategy allowed preservation of the inherent anionic charges on the nanocelluloses, e.g., CNCs, that enabled high water intake, while the added polymer chains enhanced network formation.^[^ ^43^, ^44^ ^]^ Therefore, the viscosity and storage modulus were markedly enhanced and easily tuned by varying the chain length. Recently, alkyl‐chain‐anchored CNCs, which were obtained via this strategy, were used to prepare hydrogels at a low CNC loading compared to the nonmodified ones.^[^ ^43^, ^44^ ^]^ The hydrophobized CNCs could self‐assemble into hydrogels,^[^ ^43^ ^]^ or associate with other polymers.^[^ ^44^ ^]^ To date, periodate oxidation/reductive amination‐based hydrophobized nanocelluloses have not been used as composite reinforcements.

### 3.2 Grafting from Nanocellulose in Aqueous Conditions

Polymer grafting of nanocellulose is a powerful approach to guide the self‐assembly of nanocelluloses and tune the compatibility of the nanocellulose with the matrix.^[^ ^20^, ^47^ ^]^ Free‐radical polymerization is a simple method that can be performed in water. For example, cerium ammonium nitrate (CAN),^[^ ^34^, ^48^ ^]^ potassium persulfate,^[^ ^34^ ^]^ and Fenton's reagent^[^ ^49^ ^]^ can initiate polysaccharide graft copolymerization in aqueous conditions. Cerium ion chelates with adjacent hydroxyl groups and then primary free radicals are generated on an opened glucose ring through a redox reaction. Therefore, CAN initiation has a high grafting efficiency and is used extensively in contrast with the other two methods.^[^ ^48^, ^50^, ^51^ ^]^ In 2011, Littunen et al. used CAN to graft several methacrylates and acrylates on CNF surfaces in aqueous suspension at pH 1, whereas organic solvent was used to remove homopolymer during purification.^[^ ^48^ ^]^ Glycidyl methacrylate and alkyl acrylates were readily grafted on CNFs with less than 20% homopolymers.^[^ ^48^ ^]^ Methyl methacrylate (MMA) showed a median reactivity and the product contained 25–44% homopolymer with respect to the total polymer, whereas 2‐hydroxyethyl methacrylate had the lowest reactivity, and the product contained 37–82% homopolymer.^[^ ^48^ ^]^ Inspired by this pioneering work, the latest research focused on graft copolymerization of MMA on CNCs.^[^ ^49^, ^50^, ^51^ ^]^ Spinella et al. used PMMA‐grafted CNCs to reinforce PLA, which improved the thermomechanical property significantly.^[^ ^49^ ^]^ Kedzior et al. used PMMA‐grafted CNCs to reinforce the PMMA matrix, and aimed to improve interfacial compatibility.^[^ ^50^ ^]^ However, more pronounced aggregation occurred when modified CNCs were included.^[^ ^50^ ^]^ It appears that interface engineering of nanocelluloses requires more than simple surface modification. PMMA‐grafted CNCs formed a transparent film after hot pressing,^[^ ^51^ ^]^ while ball‐milled and melt‐mixed composites exhibited darker brown with increasing CNC loading.^[^ ^50^ ^]^ Also, PMMA‐grafted CNCs significantly enhanced the elastic moduli of poly(l‐lactide) (PLLA) in the glassy and rubbery state.^[^ ^49^ ^]^ Whereas, in the CNC/PMMA composites, a higher viscosity and shear modulus was observed for the unmodified CNCs compared to the PMMA‐grafted CNCs.^[^ ^50^ ^]^ Although, the PMMA‐grafted CNCs showed better interfacial compatibility with PMMA than unmodified CNCs based on contact angle measurements, but aggregation happened due to colloidal instability and grafted polymer bridging that led to poor dispersion and hence low viscoelastic properties. Hydrochloric‐^[^ ^49^ ^]^ and sulfuric‐^[^ ^50^, ^51^ ^]^ acid‐hydrolyzed CNCs could be grafted through CAN initiation, although these two CNCs have a different surface chemistry. In contrast to CNF, which is covered with hemicellulose, CNCs contain no hemicellulose. It is necessary to study the effect of surface composition and charges on graft copolymerization of nanocelluloses through CAN initiation.

**Figure 3:**
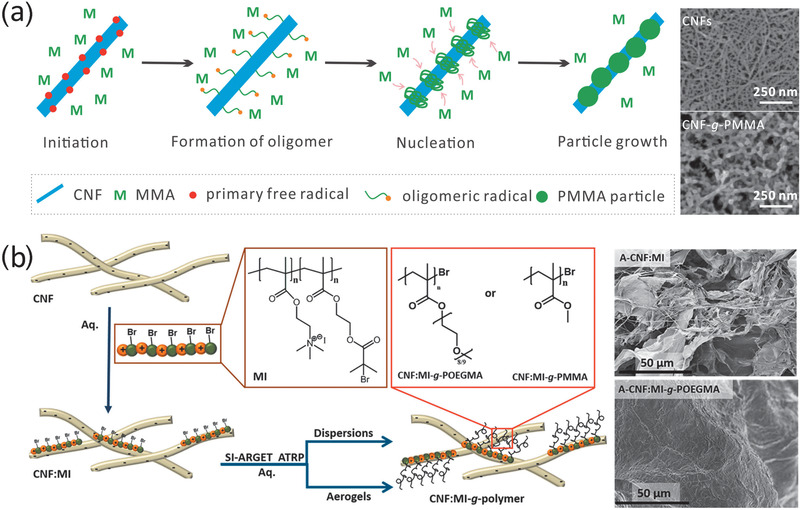
a) Grafting from CNFs via UV grafting. SEM images represent unmodified CNFs (upper) and grafted CNFs (bottom). Reproduced with permission.^[^ ^52^ ^]^ Copyright 2019, Royal Society of Chemistry. b) Grafting from CNFs via SI‐ATRP. SEM images represent CNF:MI aerogel (upper) and grafted CNF aerogel (bottom). Reproduced with permission.^[^ ^54^ ^]^ Copyright 2019, American Chemical Society.

Another graft copolymerization method is to irradiate CNF suspension with ultraviolet (UV) light in the absence of initiator.^[^ ^52^ ^]^ The authors grafted several acrylamides, methacrylates, and acrylates on CNFs, including hydrophilic and hydrophobic monomers. The weight ratio of grafted polymer to CNFs reached as high as 4.84. Different nanocelluloses had different reactivities: TOCNs > CNFs > CNCs. It was assumed that primary free radicals were generated on CNFs under UV irradiation through hemicellulose chromophores or cleavage of C—O and C—C bonds of polysaccharide backbones (**Figure** **3**a). The authors call the method UV grafting, and expect that this method may help to fabricate nanocellulose composites with a nanofiber/grafted‐matrix architecture.^[^ ^52^ ^]^ However, it remains unclear whether UV grafting occurs in organic solvents. Polymer grafting of nanocelluloses via free radical polymerization occurs on the surface, which avoids surface peeling and maintains crystalline structures of nanocelluloses.^[^ ^49^, ^50^, ^52^ ^]^

Researchers have also attempted controlled graft copolymerization of nanocelluloses in water, and have focused mainly on surface‐initiated atom transfer radical polymerization (SI‐ATRP).^[^ ^39^, ^53^, ^54^ ^]^ CNCs, with anionic sulfate half‐ester groups on the surface, are dispersed in water after immobilization of the hydrophobic initiator group, α‐bromoisobutyryl bromide.^[^ ^53^ ^]^ This modification was performed in dimethylformamide, and involved a solvent‐exchange step, although, the SI‐ATRP of *N,N*‐dimethylacrylamide was performed in water with copper mediation.^[^ ^53^ ^]^ Kaldeus et al. reported a new strategy to avoid the usage of organic solvent during initiator immobilization (Figure 3b).^[^ ^54^ ^]^ The adsorption of a cationic macroinitiator on CNFs was achieved via electrostatic adsorption.^[^ ^54^ ^]^ Hydrophobic and hydrophilic monomers could be polymerized in water owing to the amphiphilic nature of the macroinitiator.^[^ ^54^ ^]^ This method is interesting, although the macroinitiator design is complex. To date, polymer‐grafted nanocelluloses via SI‐ATRP have not been used as a reinforcing agent in composites.

Polymer grafting of nanocelluloses in water remains challenging, particularly for CNFs with an intricate structure. The purpose of the aqueous reaction is to avoid organic solvent usage. Thus, organic solvent‐free pre‐ and posttreatment is extremely important. UV grafting and aqueous SI‐ATRP exhibit promising potential.

### 3.3 Surface Coating of Nanocellulose in Aqueous Conditions

**Figure 4:**
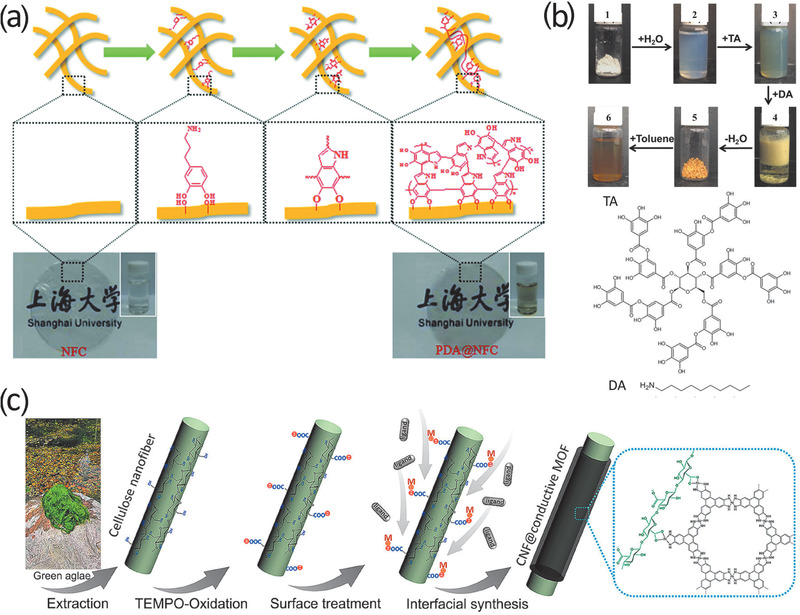
a) Schematic representation of coating PDA on CNFs. Reproduced with permission.^[^ ^56^ ^]^ Copyright 2017, Royal Society of Chemistry. b) Coating TA on CNCs at various steps: (1) CNC dry powder, (2) CNCs in water (2 wt%), (3) CNC‐TA in water (2 wt%), (4) CNC‐TA‐DA in water, (5) CNC‐TA‐DA dry powder, and (6) CNC‐TA‐DA in toluene (2 wt%). Reproduced with permission.^[^ ^62^ ^]^ Copyright 2017, American Chemical Society. c) Schematic of synthesis procedure for TOCN@MOF hybrid nanofibers. Reproduced with permission.^[^ ^67^ ^]^ Copyright 2019, American Chemical Society.

Polydopamine (PDA) can be coated onto surfaces of various materials via self‐polymerization in aqueous conditions.^[^ ^55^ ^]^ In recent years, PDA has been used to modify nanocelluloses and to fabricate the corresponding composites.^[^ ^56^, ^57^, ^58^, ^59^, ^60^, ^61^ ^]^ In 2017, Su et al. used PDA‐coated CNFs to prepare long‐term durable films (**Figure** **4**a).^[^ ^56^ ^]^ The coating was carried out in water using dopamine hydrochloride and tris(hydroxymethyl)aminomethane at pH 8.5. Dopamine reacted with the hydroxyl groups of CNFs, which resulted in dehydration and covalent bonds. Excess reagents and physically absorbed PDA were removed by rinsing with water and centrifuging.^[^ ^56^ ^]^ No organic solvent was used during the modification process, although the self‐polymerization was complex and the authors did not mention how the complete removal of absorbed PDA was confirmed. Liu et al. reported simultaneously stiffened and strengthened PDA‐coated CNF films.^[^ ^57^ ^]^ The enhanced mechanical property was ascribed to functional groups of PDA, which acted as cross‐linking sites to form cohesive interactions between PDA and CNFs.^[^ ^57^ ^]^ PDA‐coated nanocellulose composites were developed with enhanced mechanical properties, including PVA/CNC film, soy protein resin/CNF adhesive,^[^ ^59^ ^]^ and CNF/Nafion membrane.^[^ ^60^ ^]^ PDA‐coated nanocellulose can be modified further owing to the reactivity of the catechol group in PDA. For example, thiol‐containing castor oil was tethered on PDA‐coated CNCs through the Michael addition reaction, although the second modification step was performed in ethanol rather than in water.^[^ ^61^ ^]^ In summary, PDA can be coated easily on different nanocelluloses with aqueous reaction. Therefore, solvent‐exchange tends to be skipped, and the reaction is carried out directly as a nanocellulose aqueous suspension. PDA‐coated nanocellulose composites showed improved and new features.

Tannic acid (TA), which is a low‐cost natural polyphenol, can be coated on the nanocellulose surfaces. In 2017, Hu et al. first modified CNCs with TA as the primer, followed by covalent attachment of decylamine (DA) through Schiff base formation and/or Michael addition in water (Figure 4b).^[^ ^62^ ^]^ The hydrophobicity improved from 21° to 74° after decylamide group introduction.^[^ ^62^ ^]^ Phase separation occurred and modified CNCs floated above the liquid phase, which facilitated purification and dewatering.^[^ ^62^ ^]^ The dried sample could be well dispersed in organic solvent, and it was important to use dried sample as a reinforcing agent.^[^ ^62^ ^]^ Inspired by this pioneering work, TA‐coated CNCs were used to reinforce hydrogels.^[^ ^63^, ^64^ ^]^ Shao et al. fabricated poly(acrylic acid) networks in a TA‐coated CNC suspension through in situ polymerization, followed by ionic cross‐liking with Al^3+^.^[^ ^63^ ^]^ Carboxyl groups on the poly(acrylic acid) and catechol groups on the TA could form dynamic coordination bonds, which enabled self‐healing of the resultant hydrogels.^[^ ^63^ ^]^ However, the authors did not report the effect of TA coating on the reinforcement. In addition to CNCs, CNFs were also modified with TA and octadecylamine to reinforce the polypropylene–polyethylene copolymer.^[^ ^65^ ^]^ The authors observed that modified CNFs dispersed better in the polymer matrix, however, this modification did not exhibit a higher reinforcement than that with a compatibilizer.^[^ ^65^ ^]^ Compared with PDA, TA is cheaper, whereas the PDA coating is denser.

In addition to PDA and TA coating, the interfacial synthesis of metal–organic frameworks (MOFs) on nanocelluloses can be conducted in aqueous conditions.^[^ ^66^, ^67^, ^68^ ^]^ Recently, Zhou et al. coated conductive MOF layers on TOCNs to form TOCN@MOFs, in which TOCNs served as substrates for MOF growth (Figure 4c).^[^ ^67^ ^]^ A solution of Ni(OAc)_2_·4H_2_O was added to a TOCN suspension, followed by addition of 2,3,6,7,10,11‐hexahydroxytriphenylene or 2,3,6,7,10,11‐hexaaminotriphenylene.^[^ ^67^ ^]^ The reaction was performed at 80 °C for 12 h, and the product was washed with deionized water and acetone.^[^ ^67^ ^]^ The difficulty in processing MOFs was overcome by using TOCNs as substrates. MOF micropores (≈1.5 nm) and mesopores (15–50 nm) in interfibrillar cavities lead to a hierarchical micromesoporosity. The TOCN@MOFs, which can be used as flexible energy storage devices, integrate the excellent mechanical properties of the TOCNs and the high MOF electrical conductivity.^[^ ^67^ ^]^ Another TOCN@MOF was prepared by a one‐pot method to print 3D printable hydrogel inks.^[^ ^68^ ^]^ Similar to polymer grafting of nanocelluloses, surface coating of PDA, TA, and MOFs on nanocelluloses has little effect on crystallinity of nanocelluloses.^[^ ^61^, ^62^, ^64^, ^67^ ^]^ On the other hand, the optical transparency is generally sacrificed, ascribed to dark‐colored features of PDA, TA, and MOFs.^[^ ^56^, ^57^, ^58^, ^61^, ^62^, ^63^, ^64^, ^67^ ^]^ Although, the information on the rheological properties of surface coated nanocelluloses is scarce, but are reported for their ionic composite hydrogels. In general, these hydrogels showed shear thinning behavior and the storage modulus increased with the increase of modified nanocellulose content.^[^ ^64^, ^68^ ^]^

**Figure 5:**
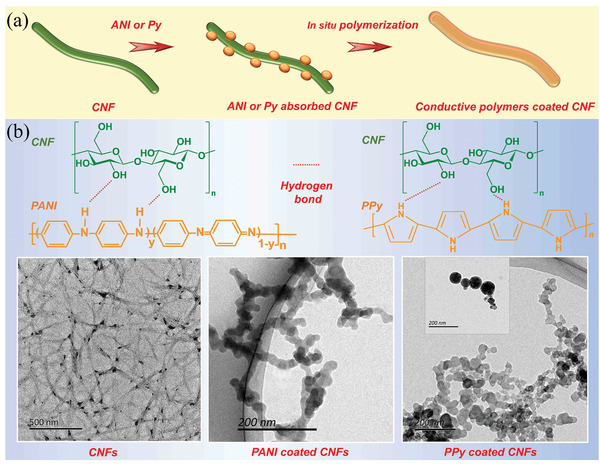
a) Schematic to illustrate the in situ polymerization of conductive polymers onto CNF. b) Demonstration of the construction mechanism and TEM images of original and PANI‐coated CNFs [Reproduced with permission.^[^ ^70^ ^]^ Copyright 2019, Elsevier], and, demonstration of the construction mechanism and TEM image of PPy‐coated CNFs [Reproduced with permission.^[^ ^71^ ^]^ Copyright 2018, American Chemical Society].

Interestingly, conductive polymers, such as polyaniline (PANI) and polypyrrole (PPy), can also be coated on nanocellulose surfaces in aqueous conditions (**Figure** **5**a). This approach combines the conductive nature of the conducting polymers with the biotemplate role of CNF, which serves as a stable and robust nanocarrier to facilitate the dispersibility of conductive polymers in water due to its excellent dispersibility and inherent nanoscale dimension.^[^ ^69^ ^]^ Because of the intermolecular hydrogen bonding between numerous hydroxyls on the CNFs and amine groups of aniline (ANI) or N—H in the pyrrole (Py) ring, PANI or PPy can be uniformly coated on the surface of CNFs by in situ polymerization to form a core–shell‐structured conductive nanocomposties (Figure 5b).^[^ ^70^, ^71^ ^]^

**Figure 6:**
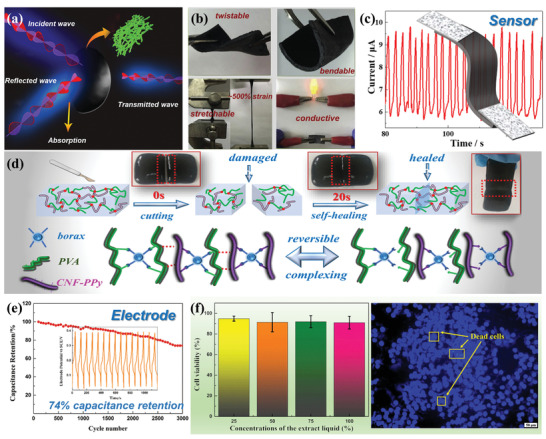
a) Schematic illustration of the EMI shielding interference of PANI/CNF flexible nanopaper. Reproduced with permission.^[^ ^72^ ^]^ Copyright 2018, American Chemical Society. b) Demonstration of the properties of conductive CNF–PANI/NR elastomers. c) Photographs showing the hydrogel‐based strain sensors attached on index fingers for recording the bending movements of fingers. b,c) Reproduced with permission.^[^ ^69^ ^]^ Copyright 2019, Elsevier. d) Schematic illustration of in situ self‐healing and dynamic reversible cross‐links of hydrogels. Reproduced with permission.^[^ ^71^ ^]^ Copyright 2018, American Chemical Society. e) Electrochemical properties of the hydrogel‐based electrode. Reproduced with permission.^[^ ^70^ ^]^ Copyright 2019, Elsevier. f) Biocompatibility diagram of the hydrogel. Reproduced with permission.^[^ ^71^ ^]^ Copyright 2018, American Chemical Society.

For example, Gopakumar et al. used the PANI‐coated CNFs to fabricate flexible, lightweight, and highly conductive paper as a sustainable microwave absorber within the 8.2‐12.4 GHz (X band).^[^ ^72^ ^]^ This cellulose nanopaper was prepared by in situ polymerization of ANI monomers on CNFs, followed by vacuum filtration of PANI/CNF aqueous suspension. The intermolecular hydrogen bonds between the amine groups of ANI monomers and the hydroxyl groups of CNFs can not only make the cellulose nanopaper more robust, but also play a vital role in shielding electromagnetic radiations. Such PANI/CNF flexible composite paper is considered a promising candidate for electromagnetic interference (EMI) shielding (**Figure** **6**a), which can be potentially used as material for flexible electrodes, sensors and paper‐based devices.^[^ ^72^ ^]^ The conductive polymer/nanocellulose composites can also be integrated into soft material platforms such as elastomer and hydrogel matrixes. Recently, Han et al. synthesized nanostructured PANI/CNF nanocomposites through in situ oxidative polymerization of ANI monomers on the surface of CNF biotemplates, which were further homogeneously dispersed into natural rubber (NR) latex to fabricate conductive CNF‐PANI/NR elastomers with a hierarchical 3D network structure.^[^ ^69^ ^]^ These stretchable conductive elastomers exhibited intrinsic flexibility, enhanced mechanical properties (tensile strength up to 9.7 MPa, Young's modulus up to 10.9 MPa), decent stretchability (elongation at break up to 511%), ideal conductivity (up to 8.95 × 10^−1^ S m^−1^), high sensitivity and good repeatability under a cyclic tensile strain (Figure 6b,c),^[^ ^69^ ^]^ demonstrating great potential for use in strain sensors to monitor the real‐time motion of human body. Apart from conductive elastomers, Han et al. further developed electroconductive hydrogels by dispersing conductive‐polymer‐decorated CNFs into the poly(vinyl alcohol) (PVA)–borax (PB) gel system.^[^ ^70^, ^71^ ^]^ Due to the dynamically reversible crosslinks formed through hydroxyl groups and borax multi‐complexation, the conductive hydrogels could be completely self‐healed within 15–20 s after mechanical damage (Figure 6d).^[^ ^71^ ^]^ By combining desirable properties of conductive polymer/CNFs nanocomposites and the PB hydrogel matrix, as‐prepared electroconductive hydrogels demonstrated high mechanical strength, stretchability and adequate conductivity, which could be practically used in flexible and self‐healing electrodes.^[^ ^70^, ^71^ ^]^ The hydrogel‐based electrodes showed a conductivity of ≈5.2 S m^−1^ and a maximum specific capacitance of 226.1 F g^−1^, and its capacitance retention was 74% after 3000 cycles (Figure 6e).^[^ ^70^ ^]^ More importantly, the hydrogel also demonstrated appealing biocompatibility, making it possible to be used as implantable bioelectronics (Figure 6f).^[^ ^71^ ^]^

In general, coating CNFs with conductive polymers in aqueous conditions is a simple and feasible method. In this approach, CNFs play three significant roles. 1) CNFs can be used as stable nanocarriers to improve the dispersing of conductive polymers in water due to their inherent nanoscale dimension and ideal dispersibility. 2) CNFs may serve as a reinforcing phase in polymer matrixes to improve their mechanical strength. For some hydrogel‐based systems, CNFs can even serve as the cross‐linking agent to bridge the 3D gel network, and the well distributed conductive polymer/CNFs are able to efficiently improve the crosslink density and establish the load‐bearing percolating structures. 3) CNFs can help to construct effective conductive paths in conductive composites because of their enabling paths for electrons and ions, providing a continuous and stable conductive network within the conductive composite materials. These advantages allow the conductive polymer‐coated CNFs to be potentially used in the emerging flexible electronics.

## 4 Enhanced Modification Approaches

Many strategies have been proposed to enhance nanocellulose modification such as enzyme‐assisted reactions and solvent‐free reactions. Karim et al. compared chemical and enzymatic methods to modify nanocelluloses in a review.^[^ ^73^ ^]^ Although the enzymatic method has some advantages, including a high specificity and mild reaction conditions, it requires further exploration.^[^ ^73^ ^]^ In this section, we focus on unconventional methods, particularly solvent‐free reactions, which enhance the modification process.

Solvent‐free nanocellulose reactions date from 2006.^[^ ^74^ ^]^ A CNC suspension was mixed with an alkyenyl succinic anhydride (ASA) aqueous emulsion, followed by filtration, freeze‐drying, and reaction at 105 °C.^[^ ^74^ ^]^ ASA acted as a reactant and as a spacer between CNCs, for which the modified CNCs could be dispersed again.^[^ ^74^ ^]^ A reaction between CNCs and octenyl succinic anhydride (OSA) with a similar process was reported recently.^[^ ^75^ ^]^ The OSA‐modified CNCs were used to form a strong gel‐like emulsion with the soy oil. The magnitude of the moduli of the emulsions stabilized by OSA‐modified CNCs was remarkably higher than that of the counterparts by unmodified CNC indicating that the OSA‐modification remarkably facilitated the formation of the strong gel‐like emulsion. Freeze‐dried CNCs were esterified by using acetic anhydride as reactant and citric acid as catalyst.^[^ ^76^ ^]^ No additional solvent was added and the reaction was performed at 120 °C.^[^ ^76^ ^]^ Transesterification between CNCs and canola oil fatty acid methyl ester occurred at 100–120 °C in the absence of solvent.^[^ ^77^ ^]^ Benzoic acid was attached to CNCs through esterification at 130 °C, which has rarely been reported for nanocelluloses.^[^ ^78^ ^]^ The hydrophobized CNCs were used to reinforce PLA, which provided a greater reinforcement, and a less viscous and more dynamic elastic behavior than that of pristine CNCs due to the better interfacial bonding with PLA.^[^ ^78^ ^]^

In 2016, Yoo and Youngblood proposed an interesting approach to graft PLA oligomer from CNCs.^[^ ^79^ ^]^ A mixture of CNC aqueous suspension and dl‐lactic acid syrup was heated at 180 °C, during which water was distilled and PLA oligomer was formed.^[^ ^79^ ^]^ Partial PLA oligomer was grafted on CNCs to form an intermediate product that was dispersed by PLA homopolymer and unreacted lactic acid.^[^ ^79^ ^]^ The modified CNCs could be esterified with fatty acids to increase the wettability in organic solvents.^[^ ^79^ ^]^ In contrast to CNCs, limited enhanced CNF modification has been reported, most likely because of the intricate CNF structures. One example is to use concentrated CNF cake as a reactor.^[^ ^80^ ^]^ After filtering a dilute CNF suspension, the obtained CNF cake had layered structures and nanochannels.^[^ ^80^ ^]^ When a reactant mixture passed through the cake, the water inside was replaced, whereas the reaction occurred at the same time.^[^ ^80^ ^]^ In this study, the solvent‐exchange process was enhanced and the CNF concentration was high, which improved the modification efficiency.^[^ ^80^ ^]^

It is concluded that the modification process can be enhanced to address the relatively low reactivity of hydroxyl groups on nanocelluloses. Solvent‐free processes, which tend to be performed at a high temperature, improve the reaction rate and present a green route.^[^ ^74^, ^75^, ^76^, ^77^, ^78^, ^79^ ^]^ Although enhanced conditions may induce bulk reactions of nanocelluloses, most researches point out that severe conditions do not significantly alter crystalline regions within a low degree of substitution.^[^ ^74^, ^77^, ^79^, ^80^ ^]^ In contrast, it is reported that crystallinity of nanocelluloses may be increased with enhanced conditions owing to hydrolysis or crystallization of amorphous regions.^[^ ^79^, ^80^ ^]^ CNCs were used most frequently, because freeze‐dried CNCs can be redispersed and the CNC viscosity is lower than CNFs and TOCNs. In some cases, modified CNCs were dried in an oven for further use.^[^ ^75^, ^77^, ^79^ ^]^ Most research has focused on the modification process, whereas limited reports exist on the corresponding composites.^[^ ^78^ ^]^

## 5 Stage‐Oriented Modification of Nanocelluloses

**Figure 7:**
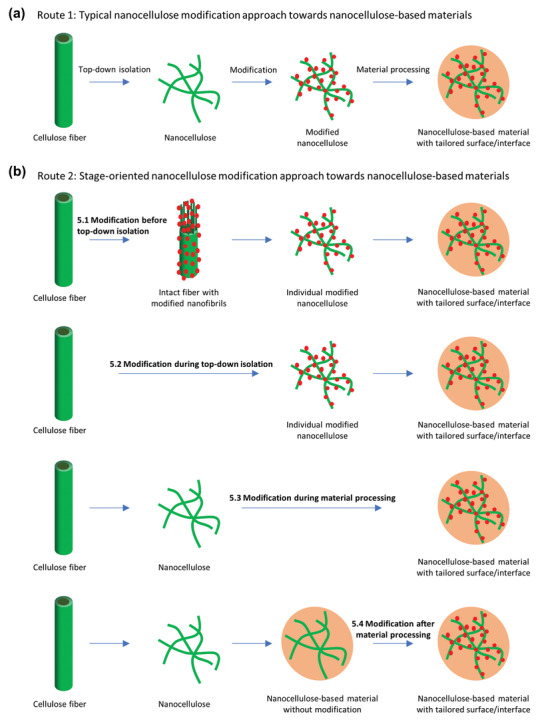
Modification of nanocelluloses with different routes. a) Typical and b) stage‐oriented modification approach towards nanocellulose‐based materials.

For most chemical modification of nanocelluloses, the reactions are performed after “top‐down” processing (isolation of CNFs, TOCNs, and CNCs) and before material processing (Route 1, **Figure** **7**a). Modification in different stages can provide new strategies to manufacture nanocellulose‐based materials (Route 2, Figure 7b). In this section, nanocellulose modification before or during “top‐down” processing is discussed (Sections 5.1 and 5.2). Modification can also be carried out during composite processing or after material formation (Sections 5.3 and 5.4). We term these strategies stage‐oriented modification of nanocelluloses.

### 5.1 Modification before Top‐Down Isolation

In theory, cellulose fibers can be modified prior to top‐down isolation, which addresses the problems caused by high nanocellulose viscosity. In 2010, Jonoobi et al. acetylated and then nanofibrillated kenaf fibers mechanically in aqueous dispersion to obtain CNFs with 5 to 50 nm diameters and hydrophobic surfaces.^[^ ^81^, ^82^ ^]^ Compared with nanocelluloses, cellulose fiber modification was carried out more readily. It was also hypothesized that the swelling of kenaf fibers during acetylation would facilitate nanofibrillation.^[^ ^81^, ^83^, ^84^ ^]^ Epoxy‐group tethered CNFs were obtained through mechanical nanofibrillation of pretreated cellulose fibers.^[^ ^85^ ^]^ Acetylated nanocellulose could also be produced by mechanical grinding of microcrystalline cellulose.^[^ ^86^ ^]^ The above modified cellulose fibers were nanofibrillated in water, and nanofibrillation in organic solvent has also been reported.^[^ ^87^ ^]^ Alkyl ketene dimer‐treated hardwood bleached Kraft pulp was disintegrated with an ultrasonic homogenizer in tetrahydrofuran.^[^ ^87^ ^]^ Approximately 30% of the product could be filtered through a glass filter with a pore size of 40 µm.^[^ ^87^ ^]^ For nanofibrillation in organic solvent, it is probably better to use a closed system with a corrosion resistance to organic solvent, such as ball milling.

In addition to modification with small molecules, polymer grafting of cellulose fibers prior to disintegration is also possible owing to the unique cellulose fiber pore structures.^[^ ^88^, ^89^ ^]^ In 2015, Tang et al. amidated TEMPO‐oxidized pulp with poly(ethylene glycol) (PEG) chains followed by disintegration.^[^ ^88^ ^]^ In 2018, Eksiler et al. grafted polycaprolactone (PCL) from the surfaces of dried oil palm mesocarp fibers followed by disintegration in the presence of ionic liquid.^[^ ^89^ ^]^ For both cases, grafted polymer chains were maintained after disintegration.^[^ ^88^, ^89^ ^]^ It is expected that this strategy may help to fabricate nanocellulose/grafted polymer composites simply.

**Figure 8:**
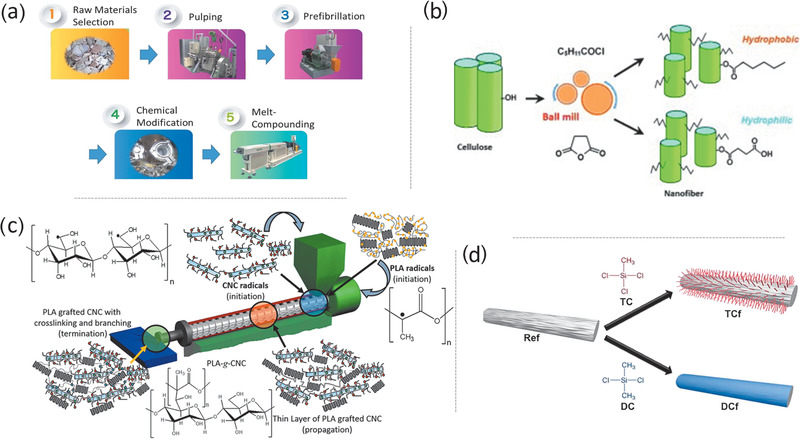
The representative processes stage‐oriented modification of nanocelluloses. a) Modification before top‐down isolation: nanofibrillation of alkenyl succinic anhydride treated pulp via melt‐extrusion. Reproduced with permission.^[^ ^90^ ^]^ Copyright 2018, Elsevier. b) Modification during top‐down isolation: esterification CNFs during nanofibrillation via ball milling. Reproduced with permission.^[^ ^99^ ^]^ Copyright 2018, Wiley‐VCH. c) Modification during composite processing: grafting PLA on CNCs via reactive extrusion. Reproduced with permission.^[^ ^112^ ^]^ Copyright 2016, Elsevier. d) Modification after material formation: modification of wet‐spun CNF filaments via CVD. Reproduced with permission.^[^ ^134^ ^]^ Copyright 2018, American Chemical Society.

Modification before the top‐down isolation strategy has been used to prepare composites, including thermoplastics,^[^ ^82^, ^85^, ^90^ ^]^ foam,^[^ ^86^ ^]^ and nanopaper.^[^ ^88^ ^]^ In 2018, Yano and co‐workers reported an integrated process (termed pulp direct kneading), in which alkenyl succinic anhydride treated pulp was nanofibrillated and dispersed simultaneously with high‐density polyethylene (HDPE) via melt‐extrusion (**Figure** **8**a).^[^ ^90^ ^]^ A high nanofibrillation degree and good reinforcement were achieved.^[^ ^90^ ^]^ This technique was used to produce the soles of running shoes (GEL‐KAYANO), which has been commercialized. However, compared with the reinforcement of neat nanocellulose, modification before top‐down isolation does not guarantee an obvious improved reinforcement.^[^ ^82^, ^86^ ^]^

Cellulose fiber modification prior to nanofibrillation is an effective approach to obtain modified nanocelluloses. Modification avoids the high nanocellulose viscosity. The reaction for cellulose fibers can be performed at a relatively high concentration.^[^ ^91^ ^]^ Dried cellulose fibers can be modified with a solvent‐free process, in which reactant is used to disperse cellulose^[^ ^81^, ^82^, ^86^, ^87^, ^92^ ^]^ or a gas‐phase reaction is performed.^[^ ^89^ ^]^ Rojas and co‐workers proposed that the heterogeneous acetylation of wood fibers could weaken the interfibrillar hydrogen bonds, which facilitates nanofibrillation.^[^ ^83^, ^93^ ^]^ However, it remains unclear how the modification affects nanofibrillation. Also, it is necessary to compare the nanofibrillation of modified and unmodified cellulose fibers in different solvents.

### 5.2 Modification during Top‐Down Isolation

Another modification approach is to integrate the modification and top‐down isolation into one step, that is, modification during top‐down isolation. In 2009, Braun et al. esterified CNCs during acid hydrolysis of cotton linter.^[^ ^94^ ^]^ Later, esterified CNCs that were obtained by a similar strategy were used to reinforce PLA, which improved the tensile strength significantly compared with PLA that was reinforced with unmodified CNCs.^[^ ^95^ ^]^ Different organic acids, such as oxalic acid dihydrate and citric acid, were used to esterify and hydrolyze cellulose simultaneously, resulting in functionalized CNCs with high yield.^[^ ^96^ ^]^ Besides esterification, it is difficult to integrate acid hydrolysis and other reactions into one step to produce modified CNCs.^[^ ^97^ ^]^ Instead, it may be possible to use weak acid, such as citric acid, to produce CNCs.^[^ ^98^ ^]^

Modification during top‐down isolation is applied more widely to CNFs. In 2012, Huang et al. developed an effective mechanochemical method to esterify CNFs during ball milling (Figure 8b).^[^ ^99^ ^]^ They used dried cellulose powders as raw material, hexanoyl chloride as a reactant, and *N,N*‐dimethylformamide as a solvent.^[^ ^99^ ^]^ The extent of substitution of obtained CNF was estimated as 0.60 after 24 h of milling, whereas the diameter was as low as 20 nm.^[^ ^99^ ^]^ Ball milling has a good resistance to organic solvents and is suitable for closed circuit grinding. Therefore, modification and nanofibrillation could be performed in various organic solvents, including *N,N*‐dimethylformamide,^[^ ^99^, ^100^, ^101^, ^102^, ^103^ ^]^ toluene,^[^ ^101^ ^]^ and dimethyl sulfoxide.^[^ ^104^ ^]^ Different reactants were used to react with hydroxyl groups of cellulose, including hexanoyl chloride,^[^ ^99^, ^102^, ^105^ ^]^ succinic anhydride,^[^ ^99^, ^104^ ^]^ acetyl chloride,^[^ ^100^, ^103^ ^]^ pentafluorobenzoyl chloride,^[^ ^101^ ^]^ and n‐dodecyl succinic anhydride.^[^ ^104^ ^]^ However, the temperature is difficult to control for reactions via ball milling.^[^ ^106^ ^]^ The milling process is generally time‐consuming and the tedious purification after reaction has not been addressed. The reaction and nanofibrillation processes must be balanced by optimizing many parameters such as solvent type, reactant concentration, ball size, milling time, and speed.^[^ ^107^ ^]^ Different types of machines may have different suitable parameters to obtain the same products.

Fu and co‐workers composited hydrophobically modified CNFs that were obtained by the above method of modification during top‐down isolation, with biodegradable polymers, including poly(propylene carbonate) (PPC),^[^ ^100^ ^]^ poly(butylenesuccinate) (PBS),^[^ ^103^ ^]^ and PCL.^[^ ^105^ ^]^ PPC is a biomaterial with a good biodegradability and biocompatibility but with a poor mechanical strength and thermal stability.^[^ ^108^ ^]^ When acetified CNFs was used, the CNFs acted as a physical cross‐linker and strong interfacial interactions were confirmed.^[^ ^100^ ^]^ CNFs reinforced the mechanical strength and enhanced the scratch resistance and scratch self‐healing behavior.^[^ ^100^ ^]^ In addition to PPC, PBS is a commercial biodegradable polymer with a high processability, high thermal and chemical resistance, and low mechanical strength.^[^ ^109^ ^]^ CNF/PBS composite fiber was obtained by melt spinning, although the CNF content was low (0.1%–0.5%).^[^ ^103^ ^]^ The authors ascribed the great reinforcement to the enhanced orientation and dispersion of CNFs and the formation of a nanohybrid shish‐kebab superstructure.^[^ ^103^ ^]^

Besides ball milling, Sakakibara et al. developed an approach to nanofibrillated wood pulp fibers during melt‐compounding, in which a diblock copolymer was adsorbed tightly onto the CNF surfaces.^[^ ^110^ ^]^ Ball milling remained the most effective tool for modification during top‐down isolation.

### 5.3 Modification during Composite Processing

The nanocellulose reaction can be conducted during composite processing via reactive extrusion, in which an extruder is used as a continuous reactor. In 2015, Wei et al. grafted polyhydroxybutyrate (PHB) onto cellulose fibers with dicumyl peroxide (DCP) as the initiator via reactive extrusion.^[^ ^111^ ^]^ It is not certain whether the cellulose fibers were nanofibrillated during extrusion. This method is also applicable to nanocelluloses, although only CNC‐based composites have been reported.^[^ ^112^, ^113^, ^114^, ^115^, ^116^, ^117^ ^]^ In 2016, Dhar et al. reported a PLA‐grafted CNC (CNC‐g‐PLA) film via reactive extrusion (Figure 8c).^[^ ^112^ ^]^ DCP‐coated PLA granules were mixed with freeze‐dried CNCs, followed by extrusion at 180 °C in a twin screw extruder.^[^ ^112^ ^]^ The gel yield was as high as 74.2% when 1 wt% CNCs was added. It was suggested that peroxide radicals reacted with PLA and CNCs to form PLA and CNC macroradicals via hydrogen abstraction.^[^ ^112^ ^]^ A thin layer of PLA was grafted onto the CNC surfaces, which improved the interfacial compatibility between PLA and CNCs.^[^ ^112^ ^]^ As a result, the tensile strength and Young's modulus were improved by 41% and 490%, respectively, when 1 wt% CNCs was added via reactive extrusion.^[^ ^112^ ^]^ In contrast, a 14% and 100% improvement was reached in the absence of DCP.^[^ ^112^ ^]^ The authors used this strategy to produce PLA/CNC films for food packaging applications, aimed at a commercial scale.^[^ ^113^ ^]^ Recently, several biodegradable polymers, including poly(butylene adipate‐*co*‐terephthalate) (PBAT) and poly(3‐hydroxybutyrate‐hydroxyvalerate) (PHBV), were reinforced with CNCs via reactive extrusion.^[^ ^114^, ^116^, ^117^ ^]^ Zheng et al. compared three processes: direct compounding, reactive extrusion using a coupling agent, and reactive extrusion using a peroxide free‐radical initiator.^[^ ^116^ ^]^ It was concluded that only the last process resulted in an improvement in strength by 13%.^[^ ^116^ ^]^ This result reminds us that nanocellulose reinforcement provides more than interfacial compatibility and this mechanism requires further exploration.

Reactive extrusion is promising because a polymer matrix can be tethered covalently on nanocelluloses in the solid state without solvent use. To date, TOCNs and CNFs have not been modified via reactive extrusion, probably because dried TOCNs and CNFs are hardly redispersed in polymer matrix. It may be possible to combine wet feeding^[^ ^118^ ^]^ and reactive extrusion. Besides reactive extrusion, nanocellulose modification during composite processing has hardly been reported. It is expected that nanofibrillation and modification can be integrated into a reactive extrusion process.^[^ ^90^ ^]^

### 5.4 Modification after Material Formation

Some nanocellulose‐based material surfaces, such as nanopapers (also referred to as sheets or films), aerogels, and filaments, can be modified after material formation. Compared with nanocellulose, the surface areas of the corresponding materials were reduced and the reaction could be performed without solvent.

In 2012, Chun et al. reported a solution‐immersion method to modify CNF film.^[^ ^119^ ^]^ The CNF film was prepared by vacuum filtration of the CNF suspension followed by hot pressing. The film was immersed in anhydrous ethanol that contained perfluoroctyltriethoxysilane as a reactant. Silanization occurred at room temperature and the excess reactant was removed by ethanol washing. A hydrophobic monolayer on the film surface was generated through self‐assembly during heat treatment, which resulted in a high water contact angle of 130.1°.^[^ ^119^ ^]^ Silane agent was used to modify the CNF film surface owing to its high reactivity with hydroxyl groups on cellulose.^[^ ^120^, ^121^, ^122^, ^123^ ^]^ Ifuku and Yano immersed CNF sheets in silane coupling agent solution for modification followed by composite preparation.^[^ ^120^ ^]^ Because of the improved compatibility between the nanofibers and matrix, the stiffness, strength, and toughness increased simultaneously after CNF sheet surface treatment.^[^ ^120^ ^]^ Multistep modification can be conducted easily by using this solution‐immersion method.^[^ ^121^, ^124^, ^125^ ^]^ Poly(*N*‐isopropylacrylamide) (PNIPAM) chains have been grafted onto a TOCN membrane.^[^ ^124^ ^]^ Cellulose ester was coated on the CNF film surfaces to adjust the surface property, which is a simple method.^[^ ^126^ ^]^ This modification method of solution‐immersion is also applicable to hydrogels and aerogels.^[^ ^127^, ^128^, ^129^ ^]^ Córdova and his team modified CNF aerogel via catalytic organo‐click chemistry,^[^ ^130^ ^]^ which is a versatile method for modification of nanocelluloses.^[^ ^131^ ^]^ Chemical vapor deposition (CVD) can be used for aerogel modification.^[^ ^132^, ^133^ ^]^ Jiang and Hsieh modified CNF aerogels with triethoxyl(octyl)silane via CVD, which made the aerogel hydrophobic and improved wet mechanical properties in water.^[^ ^133^ ^]^ In addition to films and aerogels, Cunha et al. modified wet‐spun CNF filaments with organosilanes via CVD (Figure 8d).^[^ ^134^ ^]^ Interestingly, the use of trichloromethylsilane resulted in 3D, hairy‐like assemblies on the filament surface, whereas dimethyldichlorosilane yielded continuous homogeneous coating layers.^[^ ^134^ ^]^ After modification, the surface energy was reduced and water absorption was hindered. Therefore, the wet strength of the modified filament was more than twice that of the unmodified sample.^[^ ^134^ ^]^

Modification after material formation, which is also referred to as postmodification,^[^ ^134^ ^]^ is a powerful method to modify the surface chemistry of nanocellulose‐based materials, mainly films, aerogels, and filaments. The modification can be conducted by several facile approaches, such as solution‐immersion and CVD. The wet strength can be improved with a simple modification process. For nanocellulose/hydrophilic polymer composites, this post‐modification method may be a good way to adjust the surface chemistry and broaden their application, which has not been studied widely.

### 5.5 Perspective of Stage‐Oriented Modification of Nanocelluloses

Representative processes of the above four strategies are illustrated in Figure 8 to provide an overall view of the stage‐oriented modification of nanocelluloses. These processes simplify the modification of nanocelluloses with a novel route (Figure 7) and reduce the solvent and energy consumption. The interface interactions or surface chemistry of nanocellulose‐based materials were adjusted successfully. Additional features were obtained via these interfaces or surface engineering, including an improved wet strength,^[^ ^102^, ^134^ ^]^ oil/water separation,^[^ ^132^, ^133^, ^135^ ^]^ CO_2_ adsorption,^[^ ^122^ ^]^ water barrier,^[^ ^102^, ^113^, ^125^, ^126^ ^]^ oxygen barrier,^[^ ^113^ ^]^ flame‐resistance,^[^ ^128^ ^]^ charge‐storage capacity,^[^ ^127^ ^]^ self‐healing,^[^ ^100^ ^]^ and shape memory.^[^ ^100^ ^]^ Because surface modification does not guarantee an enhanced reinforcement,^[^ ^82^, ^116^ ^]^ a theoretical study is required to reveal the structure–property relationship.^[^ ^115^, ^116^ ^]^ A prediction of the properties prior to material formation would be of great interest.

**Table** **2** summarizes the modification of different nanocelluloses and corresponding materials with different strategies. We hope that it may help generate new methods to bring nanocellulose‐based materials closer to commercialization. For example, we may design more TOCN‐based functional materials by TEMPO‐oxidized pulp modification followed by disintegration.^[^ ^88^ ^]^ For modification during top‐down isolation and modification during composite processing, the main tools were ball milling and reactive extrusion, respectively. Future studies may focus on the development of powerful tools. Integration of aqueous modification (Section 2) and stage‐oriented modification (Section 5) may result in an interesting process and novel materials. In addition, most modified nanocelluloses show a reinforcement effect, however, the research of stage‐oriented modification rarely exhibits higher reinforcement than typical modification methods.

**Table 2:** Stage‐oriented modification and corresponding reinforcement effect of nanocelluloses

	Modification before top‐down isolation	Modification during top‐down isolation	Modification during composite processing	Modification after material formation
CNCs		Esterified CNCs^[^ ^94^, ^95^, ^97^ ^]^	Modification of CNCs via reactive extrusion^[^ ^112^, ^113^, ^114^, ^115^, ^116^, ^117^ ^]^	/
		++PLA/CNC plastic^[^ ^95^ ^]^	++PLA/CNC^[^ ^112^, ^113^, ^115^ ^]^ +PBAT/CNC^[^ ^114^ ^]^ +PHBV/CNC^[^ ^116^, ^117^ ^]^	
TOCNs	TOCN‐g‐PEG^[^ ^88^ ^]^			TOCN‐g‐PNIPAM^[^ ^124^ ^]^
	++TOCN‐g‐PEG nanopaper^[^ ^88^ ^]^			
CNFs	Esterified CNFs^[^ ^81^, ^82^, ^84^, ^86^, ^87^, ^90^ ^]^ Epoxidated CNFs^[^ ^85^ ^]^ CNF‐g‐PCL^[^ ^89^ ^]^	Esterified CNFs^[^ ^99^, ^100^, ^101^, ^102^, ^103^, ^104^, ^105^ ^]^ Diblock copolymer adsorbed CNFs^[^ ^110^ ^]^		/
	–PLA/CNF^[^ ^82^ ^]^ ++PVA/CNF^[^ ^85^ ^]^ +PEVA/CNF foam^[^ ^86^ ^]^ ++HDPE/CNF^[^ ^90^ ^]^	+PPC/CNF^[^ ^100^ ^]^ ++CNF nanopaper^[^ ^102^ ^]^ +PBS/CNF^[^ ^103^ ^]^ +LDPE/CNF^[^ ^104^ ^]^ ++PCL/CNF^[^ ^105^ ^]^ +HDPE/CNF^[^ ^110^ ^]^		++CNF nanopaper^[^ ^119^, ^126^ ^]^ ++CNF aerogel^[^ ^127^, ^135^ ^]^ ++CNF/acrylic resin^[^ ^120^ ^]^ ++CNF filament^[^ ^134^ ^]^

Note: PEVA: poly(ethylene‐*co*‐vinyl acetate); LDPE: low‐density polyethylene; ‐: no obvious reinforcement with modified nanocelluloses; +: obvious reinforcement with modified nanocelluloses, but comparable or inferior to unmodified nanocelluloses; ++: higher reinforcement with modified nanocellulose than those with unmodified nanocelluloses.

### 5.6 Technological Applications of Surface Engineered Nanocelluloses

To realize various desired technological applications, nanocellulose can be modified based on various mechanisms and accordingly processed to diversified material platforms, such as film, gel, foam, elastomer, etc. Abundant nanocellulose‐based products can be obtained by adding different matrixes with different process design, so as to be applied to sustainable packaging, water treatment, CO_2_ capture, flexible electronics, energy storage and even biomedical tissue engineering, which is listed in **Table** **3**.

**Table 3:** Technological applications of engineered nanocelluloses

Material component		Product forms	Nanocellulose modification mechanism	Specific procedure	Technological applications	Refs.
Nanocellulose	Others					
CNFs	Potato starch	Film	Hydrogen bonds	Blending; Solvent casting	UV resistant sustainable packaging	^[^ ^136^ ^]^
CNFs	Chitin nanocrystals	Aerogel	Hydrogen bonds	Blending; Freeze‐drying	Water treatment, antibacterial; antioxidant	^[^ ^137^ ^]^
TOCN	PEI	Foam	Noncovalent interaction	Sol–gel method; Freeze‐drying	CO_2_ capture	^[^ ^138^ ^]^
CNFs	Lupamin	Membrane	Hydrogen bonds	Blending; Solvent evaporation	CO_2_ capture	^[^ ^139^ ^]^
TOCN	Amino‐silanes	Film	Grafting of aminosilanes	Impregnate	CO_2_ capture	^[^ ^122^ ^]^
CNFs	PANI; NR	Elastomer	Hydrogen bonds	In situ polymerization; Latex coagulation	Train sensors and flexible electrodes	^[^ ^69^ ^]^
CNFs	CNTs; PVA	Hydrogel	Electrostatic repulsion	Blending; Sol–gel method	Flexible supercapacitor	^[^ ^142^ ^]^
TOCN	CNTs; PANI; PVA	Hydrogel	Electrostatic repulsion	Blending; In situ polymerization; Sol–gel method	Flexible supercapacitor	^[^ ^140^ ^]^
CNCs	CNTs; PVA; PAA; PANI	Membrane	Electrostatic repulsion	Electrospinning; In situ polymerization	Flexible supercapacitor	^[^ ^141^ ^]^
CNCs	Cellulose	Mat	Noncovalent interaction	Electrospinning	Scaffold for tissue engineering	^[^ ^144^ ^]^
CNFs	Alginate	Hydrogel	Ionic cross‐linking	3D bioprinting	Tissue engineering	^[^ ^145^ ^]^

Note: Lupamins: polyvinylamine; PEI: polyethylenimine; PANI: polyaniline; NR: natural rubber; CNT: carbon nanotube; PVA: poly(vinyl alcohol); PAA: poly(acrylic acid); PPy: polypyrrole.

**Figure 9:**
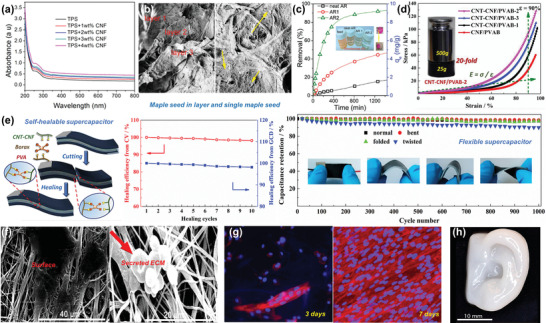
a) UV absorbance graph of starch‐CNF films. Reproduced with permission.^[^ ^136^ ^]^ Copyright 2018, Wiley‐VCH. b) FESEM images and c) Dye water treatment of aerogels. b,c) Reproduced with permission.^[^ ^137^ ^]^ Copyright 2017, Royal Society of Chemistry. d) Mechanical behavior for the gels under compression. e) Schematic illustration of the self‐healable and flexible supercapacitor. d,e) Reproduced with permission.^[^ ^142^ ^]^ Copyright 2019, Elsevier. f) SEM and g) confocal laser scanning microscopy (CLSM) images of hDFCs cultured on the bioscaffold. f,g) Reproduced with permission.^[^ ^144^ ^]^ Copyright 2014, American Chemical Society. h) Image of 3D printed human ear. Reproduced with permission.^[^ ^145^ ^]^ Copyright 2015, American Chemical Society.

For instance, Balakrishnan et al. applied CNFs as the reinforcing phase to disperse in starch matrix to make a type of nanocellulose‐reinforced biocomposite films by solvent casting technique.^[^ ^136^ ^]^ This strategy enhanced the comprehensive properties of starch‐based films, improved the mechanical properties, decreased the water vapor permeability, increased transparency and endowed the UV shielding properties (**Figure** **9**a), making such films suitable for sustainable packaging.^[^ ^136^ ^]^ Gopi et al. further developed chitin nanocrystals decorated 3D cellulose aerogels with an innovative maple seed‐like morphology, which could be used as bio‐based green material for dye adsorption from aqueous solution (Figure 9b,c).^[^ ^137^ ^]^ Interestingly, the excellent enhanced anti‐bacterial and anti‐oxidant properties made these aerogels easy to be produced on an industrial scale for commercial applications.^[^ ^137^ ^]^ In addition, Sehaqui et al. and Ansaloni et al. combined nanocellulose with polyethylenimine (PEI) and Lupamin, respectively, by noncovalent interaction to prepare CO_2_ adsorbent materials.^[^ ^138^, ^139^ ^]^ Subsequently, Valdebenito et al. provided a simple and straightforward method with single CNFs films followed by grafting of the aminosilanes to produce CO_2_ adsorbent materials.^[^ ^122^ ^]^ These films with thermal stability showed good adsorption of CO_2_ as subjected to 99.9% CO_2_ flow at 25 °C.^[^ ^122^ ^]^ In addition, nanocellulose‐based products are also widely used in the field of flexible electronics.^[^ ^69^, ^70^, ^71^, ^140^, ^141^, ^142^ ^]^ Nanocellulose plays the role of nanotemplate as the carrier of conductive materials such as PANI, PPy, and carbon nanotubes (CNTs), promoting the uniform dispersion of conductive materials and contributing to the establishment of a stable conductive network.^[^ ^69^, ^70^, ^71^, ^140^, ^141^, ^142^ ^]^ For example, Han et al. carried CNTs with CNF templates and dispersed CNT/CNF nanohybrids into PVA matrix to fabricate dynamically cross‐linked electroconductive hydrogels.^[^ ^142^ ^]^ The gel‐based electrodes exhibited high compression stress (≈93 kPa) (Figure 9d), high conductivity (10.0 S m^−1^) and 20 s self‐healing capability. The assembled self‐healable and flexible supercapacitors (Figure 9e) with a specific capacitance of 117.1 F g^−1^ exhibited a capacitance retention of 98.2% after 10 damage/self‐healing cycles, and a capacitance retention of 95% after 1000 cycles under various deformation.^[^ ^142^ ^]^ To improve the electrochemical properties, PANI layer can further cover nanocellulose‐stabilized CNTs (nanocellulose/CNT) to form a core–shell‐structured conductive hybrids (PANI@ nanocellulose/CNT).^[^ ^140^, ^141^ ^]^ In addition to sol–gel method, the PANI@ nanocellulose/CNT conducting material can also be obtained by electrospinning for flexible supercapacitors.^[^ ^141^ ^]^ Due to the good biocompatibility and similar mechanical properties to extracellular matrix, nanocellulose‐based biomaterials can serve as an excellent bioscaffold to cells adhesion and growth, promoting their development as a substitute medical biomaterial.^[^ ^143^ ^]^ He et al. reported an all‐cellulose scaffold material (cellulose/CNC) for tissue engineering with a directional CNC structure. The cultured cells (primary human dental follicle cells, hDFCs) could proliferate rapidly not only on the surface but also deep inside the bioscaffold (Figure 9f).^[^ ^144^ ^]^ More importantly, the aligned nanofibers of cellulose/CNC mat exhibited a strong effect on directing cellular organization (Figure 9g).^[^ ^144^ ^]^ Markstedt et al. developed a bioink composed of CNFs with outstanding shear thinning properties and alginate with fast cross‐linking ability for 3D bioprinting of living soft tissue with cells (Figure 9h).^[^ ^145^ ^]^

From the perspective of science and economics, nanocellulose, as a gift of nature, has great potential for packaging, water treatment, CO_2_ capture, flexible electronics and tissue engineering applications. However, there are still some questions about nanocellulose‐based products and their applications. 1) It is necessary to develop nanocellulose composite with other renewable and biodegradable materials for improving the performance, reducing the cost, and replacing the widely used synthetic resin composite materials. 2) The nanocellulose based products should be designed according to the market demand, and fortified with new properties suitable for flexible electronics, biomedical applications, and so on. 3) Regarding the application of cellular bioscaffold, the interaction mechanism between cells and nanocellulose remains unclear, and the long‐term biosecurity has not yet been systematically assessed, so such materials require further in vivo studies.

## 6 Nonmodification Interface Engineering

Although surface modification of nanocelluloses forms a compatible interface for hydrophobic matrices and imparts excellent functionalities, it is highly challenging and obscured (especially for covalent modification) as far as reinforcement is concerned, let alone the environmental issues. Because: i) the modification process often requires organic (nonpolar) media, therefore, swelling and dispersion of nanocelluloses are not enough, and hence, homogeneous modification is not possible;^[^ ^20^, ^22^ ^]^ ii) the reactivity of cellulose —OH groups is low compared with other corresponding alcohols owing to the chemical structure and steric effects produced from the supramolecular structure of cellulose;^[^ ^20^, ^146^ ^]^ iii) the availability of most reactive —OH groups on the surface (i.e., —OH groups present at C‐6 position; see Figure 1f) is limited (only one‐third for CNCs and one‐half for CNFs), since some are inserted inside the nanocellulose particles;^[^ ^22^, ^147^ ^]^ iv) the accessibility of some surface —OH groups is blocked by surface impurities absorbed during nanocelluloses processing that need extra steps to clean;^[^ ^148^ ^]^ v) if not performed carefully, modification treatments (e.g., esterification) can attack the crystalline core of nanocelluloses and reduce their reinforcing ability;^[^ ^149^ ^]^ and vi) literally, the —OH groups are important for the development of nanocellulosic materials with high mechanics through the formation of a strong interfibrillar entangled network via hydrogen bonding. In fact, the hydrogen bonds in a nanocellulose network are dynamic, i.e., they break and reform when (cyclically) strained, and therefore, are beneficial for promoting materials’ performance.^[^ ^150^ ^]^ Also, most of the noncovalent nanocellulosic materials, especially those made of hydrophilic and hydrophobic components, require involvement of chemical surfactants or compatibilizers.

Consequently, surface modification of nanocelluloses should not be overwhelmingly considered a one‐way route for the formation of good interfaces between nanocelluloses and (immiscible) matrices, and for imparting functionalities toward high‐performance and functional nanocellulosic materials. Therein, we highlight four interesting, fast, facile, and promising strategies, which rely on the physical interface engineering rather than the surface engineering of nanocelluloses in order to form strong advanced nanocellulosic materials.

### 6.1 Impregnation of Nanocellulose Preforms

**Figure 10:**
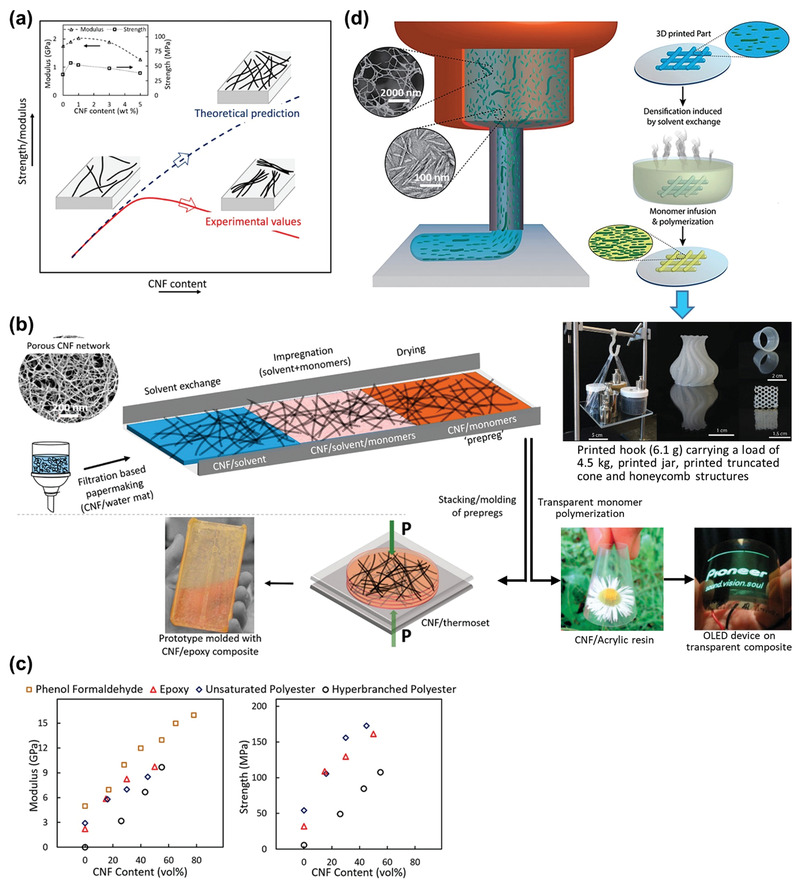
Impregnation of native nanocellulose preforms with polymers enables high performance nanocomposites with high nanocellulose share. a) Typical mechanical behavior of nanocellulose‐based nanocomposites in which the mechanics start to fall due to the nanocellulose agglomeration at high content. Reproduced with permission.^[^ ^23^ ^]^ Copyright 2018, American Chemical Society. b) Schematic of impregnation method to obtain nanocomposites with well‐dispersed high nanocellulose content. The process is similar to the paper making process, therefore, can be scaled‐up by roll‐to‐roll production of the nanocomposites and devices on them. Reproduced with permission.^[^ ^23^ ^]^ Copyright 2018, American Chemical Society. Image of the CNF/acrylic resin transparent nanocomposite reproduced with permission.^[^ ^154^ ^]^ Copyright 2005, Wiley‐VCH. Image of the OLED device on the CNF nanocomposite reproduced with permission.^[^ ^158^ ^]^ Copyright 2009, Elsevier. c) Increasing mechanical properties with increasing CNF content indicate that the CNF was well‐dispersed in the composites even at high percentages. Reproduced with permission.^[^ ^23^ ^]^ Copyright 2018, American Chemical Society. d) 3D printing of nanocellulose scaffolds/preforms with complex shapes and subsequent fabrication of nanocomposites via resin impregnation. Reproduced with permission.^[^ ^160^ ^]^ Copyright 2019, Wiley‐VCH.

A major consideration for nanocellulose‐based materials research, especially for polymer‐matrix nanocomposites, is to develop strategies that allow high nanocellulose content in order to enable environmentally friendly robust materials. A vast majority of present nanocomposites contain nanocelluloses below ≈25 wt%, primarily because the nanocomposite mechanics start to fall due to the nanocellulose agglomeration at high content (**Figure** **10**a).^[^ ^23^ ^]^ It is even true for the cases where compatibility of nanocelluloses is high with the polymer matrix. It means that even nanocelluloses are tailored to be compatible in hydrophobic matrices, they tend to agglomerate at high concentration during mixing because of their (especially of CNFs') large aspect ratio and high specific surface area that ranges from several hundred to reaching 800 m^2^ g^−1^.^[^ ^151^, ^152^ ^]^ Although, the severity of the agglomeration of native (nonmodified) nanocelluloses is lower during mixing with hydrophilic matrices in water, the fabricated materials show practically undesirable high moisture sensitivity.

In that regard, nanocomposites films with high nanocellulose content could be prepared simply by the impregnation of matrices into nanocellulose preforms, such as nanopaper, aerogel, and organogel (Figure 10b).^[^ ^153^, ^154^ ^]^ The meso‐ and nanopores (pore size may vary from 5.5 to 50 nm for nanopapers and several nanometer to ≈100 nm for aerogels, depending on the type (dimension) of nanocelluloses used and the preform preparation conditions)^[^ ^152^ ^]^ of these preforms could easily be imbibed by resins via natural capillary action without employing any special tricks. Therefore, the impregnation of resins, regardless of hydrophilic or hydrophobic, may happen quite homogeneously.

Nanocomposites films with a high nanocellulose content as high as 60 to >80 wt% were prepared by this process.^[^ ^154^, ^155^ ^]^ The nanostructure and nanocellulose content of the preforms were easily tunable, for example, by compression or solvent exchange, which enabled adaptability of the mechanical, thermomechanical, and optical properties of the nanocomposites. The mechanical properties of these nanocomposites films, though dependent on the type of resins used, were increasingly high with increasing nanocellulose content, owing to the good dispersion of nanocelluloses and the formation of intense hydrogen bonds in the preforms (Figure 10c). For example, an epoxy matrix composite contained 60–70 wt% CNFs had an astonishingly high *E* of up to 21 GPa, five times higher than that of most engineered plastics, and a high σ of up to 325 MPa, but it was as flexible and foldable as a paper.^[^ ^154^ ^]^ The nanocomposites were also optically transparent (>80%) due to the 1/10th width of the CNFs compared to the visible light wavelength.^[^ ^156^ ^]^ Another property should be emphasized that the CTE of these nanocomposites was incredibly low at 6 ppm K^−1^ only, twenty times lower than that of the neat epoxy. A low CTE on the order of 6 ppm K^−1^ is remarkable for a transparent and flexible high‐strength composite film for enabling flexible electronics. Therefore, these nanocomposites can simply be described as the unique materials that are as transparent and thermally stable as glass, as strong as steel, and as flexible as plastic. These unique nanocomposites are being considered as the substrate material for optoelectronic applications (Figure 10b).^[^ ^154^, ^157^, ^158^ ^]^

Although, it is counterintuitive to the discussion of this section, we note that further functionalization of the high‐performance nanocomposites could be done by the above described chemical processes. Importantly, impregnated nanocomposite production can easily be scaled‐up via roll‐to‐roll processing technology as the “paper‐making process” along with the potential of integration of device manufacturing steps (Figure 10b).^[^ ^23^, ^159^ ^]^ Recently, 3D‐printing technology was employed to form a nanocellulose 3D scaffold followed by impregnating it with resin, e.g., (hydroxyethyl)methacrylate resin (Figure 10d). The nanocomposites exhibited highly aligned microstructures and high mechanical properties, and allowed fabrication of nanocomposites with 3D complex shapes.^[^ ^160^ ^]^

### 6.2 Pickering‐Stabilization Strategy

**Figure 11:**
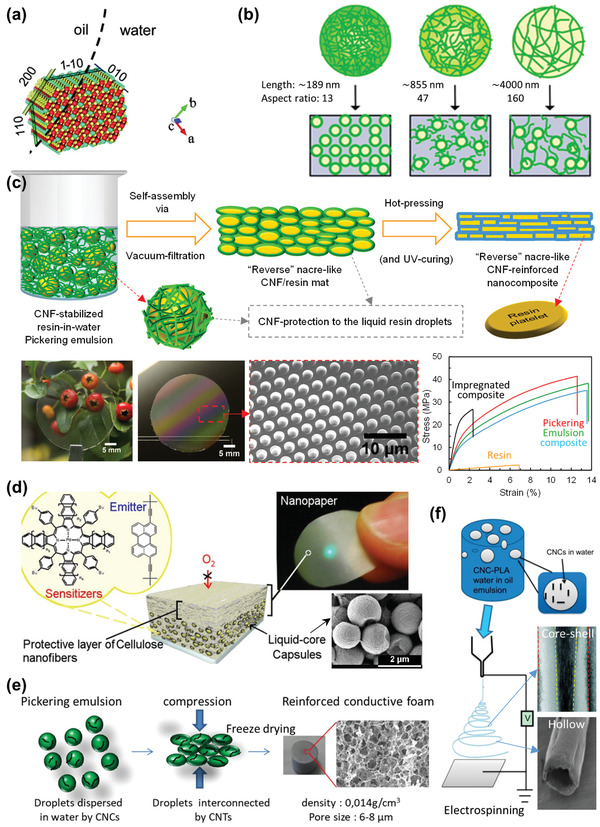
Fabrication of diverse nanocellulosic materials through interfacial assembly of nanocelluloses in Pickering emulsions. a) Schematic representation of the stabilization of the CNC (Iβ crystal) at the oil/water interface. Reproduced with permission.^[^ ^164^ ^]^ Copyright 2011, American Chemical Society. b) Schematic to show that the short CNCs promote formation of individual droplets, whereas long CNFs promote networking systems. Reproduced with permission.^[^ ^162^ ^]^ Copyright 2013, The Royal Society of Chemistry. c) Fabrication of optically transparent, low‐CTE, 3D‐moldable, strong, and tough CNF‐reinforced nanocomposite from CNF‐stabilized resin‐in‐water Pickering emulsion. Reproduced with permission.^[^ ^166^ ^]^ Copyright 2017, American Chemical Society. d) Functional photon energy upconverting nanopaper prepared by trapping sensitizer and emitter molecules into the hexadecane oil droplets having a nanocellulose shell. Reproduced with permission.^[^ ^175^ ^]^ Copyright 2014, American Chemical Society. e) Fabrication of conductive ultralight nanocellulosic foam by forming O/W Pickering emulsion stabilized by CNCs/CNTs (carbon nanotubes). Reproduced with permission.^[^ ^182^ ^]^ Copyright 2019, Elsevier. f) Nanocellulosic nano‐to‐microscale fibers produced by electrospinning of W/O CNC‐PLA emulsions. The smaller droplets produced thin CNC‐core/PLA‐shell fibers, and bigger droplets produced comparatively thicker hollow‐core fibers. Reproduced with permission.^[^ ^186^ ^]^ Copyright 2013, American Chemical Society.

Pickering stabilization of emulsion can be described as the stabilization of interfaces of two immiscible liquids by solid particles. Recent studies have shown that the nanocelluloses are exceptionally effective in stabilizing oil–water interfaces by irreversible adsorption.^[^ ^161^, ^162^, ^163^, ^164^, ^165^ ^]^ A major drive for the consideration of nanocelluloses in stabilizing emulsions includes their low toxicity and biocompatibility, therefore, could be applied in cosmetics, drug delivery, etc.^[^ ^165^ ^]^ Literally, the crystals of cellulose are not completely hydrophilic. Rather, their different crystal planes show varying affinity to water. The two most broad crystal planes in terms of surface exposure contain hydroxyl groups that are responsible for the hydrophilic property, (1−10)β/(100)α and (110)β/(010)α (**Figure** **11**a). Another two planes are located at the corners of the cellulosic crystals and have a small surface exposure. One of which is hydrophobic (200)β/(1ī0)α plane with having C—H moieties, and another is hydrophilic (010)β/(110)α plane (Figure 11a). Therefore, the reason for oil–water interface stabilization can be assigned to their amphiphilic character arising from the coexistence of more hydrophilic and less hydrophobic characters at the surface of the CNCs.^[^ ^163^, ^164^ ^]^ On the other hand, long thin CNFs with very high aspect ratio have also been studied as the Pickering emulsion stabilizer. A point to note, probably, the Pickering stabilization mechanism is different for CNFs compared to CNCs, because of their high aspect ratio and a nature to be entangled. Although CNFs are composed of CNCs, the orientation of their hydrophilic and hydrophobic planes at the oil–water interface is yet obscured. Also, CNFs are typically covered with a thin hemicellulose coating,^[^ ^19^ ^]^ which makes the interpretation of the stabilization mechanism complicated. However, it was reported that the long high‐aspect‐ratio nanocelluloses (length: ≈4 µm; aspect ratio: ≈160), i.e., CNFs, promoted the formation of interconnected network in the emulsion (Figure 11b).^[^ ^162^ ^]^ The dense network in water trapped and stabilized the oil (hexadecane) droplets without coalescence.

Using this Pickering stabilizing strategy, a uniform mixture of hydrophilic nanocelluloses and hydrophobic curable liquid resin (such as acrylic resin, polystyrene, polybutylmethacrylate, etc.) was easily formed in water (Figure 11c).^[^ ^166^, ^167^, ^168^, ^169^, ^170^, ^171^, ^172^ ^]^ The homogenous mixing was done free of any extra chemicals, such as surfactant or dispersant, just by employing a high shearing energy.^[^ ^166^, ^167^, ^168^, ^169^, ^171^ ^]^ The nanocellulose‐covered resin droplets were retrieved after removing water by casting or vacuum‐filtration like a “paper‐making process.” The obtained “raw” nanocomposite films were further processed by hot‐compression to yield highly optically transparent (≈85% @ 4–25 wt% nanocellulose) and 3D‐moldable nanocomposite films.^[^ ^166^, ^167^, ^168^, ^169^, ^171^ ^]^ The literature suggested that 3D‐moldability in several length scales (such as, in macroscale, e.g., contact lens, as well as in micro/nanoscale, e.g., polymeric microlens/nanopillar arrays, via a simple form of the “imprint lithography” or hot‐embossing technique using an oppositely patterned substrate) was exclusively facilitated owing to the presence of liquid resin droplets in the “raw” nanocomposite films.^[^ ^166^, ^168^ ^]^ Interestingly, the nanocomposites had higher σ and large strain to failure, hence, high toughness compared to that of the impregnated nanocomposites films of similar constituents. This result was due to the spontaneous formation of the hierarchical brick‐and‐mortar‐like efficient and cooperative interfaces between the resin droplets and the nanocellulose network.^[^ ^166^, ^171^ ^]^ Furthermore, by easily adjusting the droplet size during emulsion formation (e.g., by changing water content in the emulsion), the mechanical properties of the nanocomposites were tuned.^[^ ^166^, ^171^ ^]^ Remarkably, a 10 wt% CNC reinforced nanocomposite film possessed CTE of only 3–5 ppm K^−1^ in the length/width direction due to the in‐plane orientation of the deposited nanofibers.^[^ ^167^, ^168^ ^]^ Therefore, the silver nanowires deposited on these nanocomposite films exhibited low physical damage, and hence, high electrode performance even at a temperature as high as 180 °C.^[^ ^167^ ^]^ However, it was noted that the CTE of thickness direction was very high due to the in‐plane orientation of the nanofibers. Because, a CNF‐network is strong and tough in in‐plane direction, but highly flexible in out‐of‐plane direction.^[^ ^154^ ^]^ However, due to the thinness of the nanocomposite films (≈100 µm), there might be no practical problem in their potential application in optoelectronics.^[^ ^154^ ^]^ Moreover, the moldability of the nanocellulose composites in macro‐ to nanoscale enabled by the Pickering emulsion process may be advantageous in some photonic and optoelectronic applications. For instance, nano‐ and microscale surface features support strong optical resonance and reduce plasmonic loss, which can enhance and effectively control light absorption, scattering, and outcoupling to improve the performance of the optoelectronic devices such as solar cells, light‐emitting diodes (LEDs), etc.^[^ ^173^ ^]^

The Pickering‐stabilization process is facile and highly versatile for cellulose nanocomposite preparation. Instead of liquid resins, latex particles suspended in water with the assistance of surfactants were also encapsulated by the nanocellulose‐network, and strong nanocomposite films were produced.^[^ ^174^ ^]^ Therein, it can be suggested that the microparticles of engineering plastics could also be stabilized in water by the nanocellulose network, and the recipe could finally be processed into potentially high‐strength and thick structural nanocomposites with a hierarchical brick‐and‐mortar‐like microstructure. Thereby, the need for surface modification of nanocelluloses, or the use of chemical surfactants, dispersants, or compatibilizers could be overruled.

Furthermore, it was demonstrated that the functional properties could be inserted in the nanocomposite films by simply adding a functional component in the oil droplet phase. As a case example, a photon energy upconverting nanopaper was prepared by trapping sensitizer and emitter molecules into the hexadecane oil droplets (Figure 11d).^[^ ^175^ ^]^ In another example, paraffin wax microdroplets were encapsulated in the TOCN network to form a nanocomposite film with good mechanics (σ = 30 MPa and strain to failure = 12%) for storing and releasing thermal energy, which is a promising lightweight, structural and functional candidate for smart building applications.^[^ ^176^ ^]^ The film also exhibited reversible transparency due to the phase change of the wax droplets from a liquid state to a solid state. A point to be noted that owing to the formation of a dense network around the oil‐phase, the wax did not spill‐out even when it was liquid, or the oxygen permeation was very low that the sensitizer and emitter showed prolonged photon energy upconverting activity.^[^ ^175^, ^176^ ^]^ Also, by using Pickering‐stabilizing process, robust nanocellulose‐shell (ultrathin thickness of <50 nm) capsules were prepared that might find potential application in drug delivery and food applications.^[^ ^170^, ^177^ ^]^

Meanwhile, highly mechanically performed foams of which cell‐walls are reinforced by nanocelluloses received a great interest.^[^ ^178^, ^179^ ^]^ Their applications may range from food and drug delivery to purely technical.^[^ ^179^ ^]^ Pickering stabilizing nature of nanocelluloses has been suitably exploited to construct highly porous (as high as 99%), ultralow density, robust, and functional foam structures with a great variety.^[^ ^179^, ^180^, ^181^, ^182^, ^183^, ^184^, ^185^ ^]^ Also, the foam cell structure and size can be easily controlled by controlling the size of the droplets, hence, tunable mechanical properties can be readily achieved even at a similar foam density. When an oil‐in‐water (O/W) Pickering emulsion stabilized by the hydrophilic nanocelluloses was freeze‐dried, sublimation of liquids (i.e., both oil and water) happened, leaving behind a foam with the nanocellulose‐only cell walls.^[^ ^184^ ^]^ Hydrophilic, nanocellulose‐interactive polymers and nanoparticles were also added to form functional foams with nanocomposite cell walls.^[^ ^182^, ^184^, ^185^ ^]^ For instance, a conductive ultralight (density = 14 mg cm^−3^) foam was formed by adding carbon nanotubes in the emulsion that remained dispersed in the water phase along with the CNCs, and produced a composite cell wall (Figure 11e).^[^ ^182^ ^]^ These foams could find potential use in batteries, shielding materials, and conducting walls. Conversely, water‐in‐oil (W/O) Pickering emulsions were also formed using a curable resin as the oil phase and hydrophobized CNFs as the stabilizer.^[^ ^180^ ^]^ After sublimation of water, the emulsion resulted in a foam comprised of cells with the composite walls. The foams with composited cell walls typically had better compressive mechanics than the foams comprised of bare nanocellulose cell walls. Apart from Pickering emulsification route, foams were also produced by directly stabilizing air‐bubbles in water using a combination of CNFs and other additives such as lauric acid sodium salt and octylamine.^[^ ^181^, ^183^ ^]^ The stabilization mechanism of air bubbles by nanocelluloses was considered similar as to the stabilization mechanism of oil droplets. The nanocelluloses were strongly adsorbed onto the surface of the air‐bubbles and formed a layer at the interface that strongly resisted shrinkage and expansion of bubbles, minimized Ostwald ripening, and created long‐lasting stable foams. The additives gave an extra support as the surfactant. However, the compressive *E* of the resultant foams (437 kPa) from air‐water Pickering system was lower than that of the foams prepared by direct sublimation of the nanocellulose/water suspension.^[^ ^181^ ^]^

Another interesting work demonstrated that the emulsion technology could be exploited to create diverse nanocellulosic nano‐to‐microscale fibers (Figure 11f).^[^ ^186^ ^]^ It was shown that merely by changing the diameter of water/CNC droplets in the poly(lactic acid) (PLA) solution (i.e., a W/O emulsion), the ultrafine electrospun fibers assumed either a thin (400–500 nm) core–shell structure (droplet size <3 µm) or a comparatively thicker (>500 nm) hollow‐core structure (droplet size > 6 µm). In the core–shell fibers, the core was composed of aligned CNCs and shell was composed of PLA, whereas, in the hollow‐core fibers, the CNCs were aligned at the inner surface of PLA cylinder. The mechanism was mainly thought be derived by the difference in the way of the evaporation of the solvents (i.e., water and chloroform/toluene). The mats prepared from these nanocomposite fibers (mixture of 80% core–shell and 20% hollow‐core fibers) showed high σ (two times) and *E* (seven times) compared to that of the mat composed of electrospun PLA‐only fibers.

Finally, the technical and industrial aspects of the emulsion technology are already known for many decades, especially, in food, cosmetics, and pharmaceutical areas. Therefore, it can be envisaged that this facile but versatile technology will soon convey new dimensions to the nanocellulose‐based materials research and application.

### 6.3 Brick‐and‐Mortar Assembly

Recently, the strong, stiff, and tough composite structures of biological materials such as nacre, crustacean cuticles, shells, and bones have been shedding light in designing and fabricating mechanically robust, light‐weight, and truly structural composite materials.^[^ ^187^, ^188^, ^189^, ^190^ ^]^ Therein, we focus on the nacre—the mollusk shell, the natural structure of which has gained much interest as a strong, stiff, and tough model system in materials science. It has a unique synergistic strengthening, stiffening and toughening mechanism, thanks to its alternating “brick‐and‐mortar” arrangement of hard but brittle aragonite platelets (95 vol%) and soft biopolymer layers (5 vol%).^[^ ^188^ ^]^ The hard aragonite platelet layers bestow the load bearing property, whereas, the soft biopolymers (chitin and proteins) in between the platelets help stress transfer across interfaces through mutual sliding, and impede crack propagation through crack deflection and crack bridging.^[^ ^188^, ^189^, ^191^ ^]^

**Figure 12:**
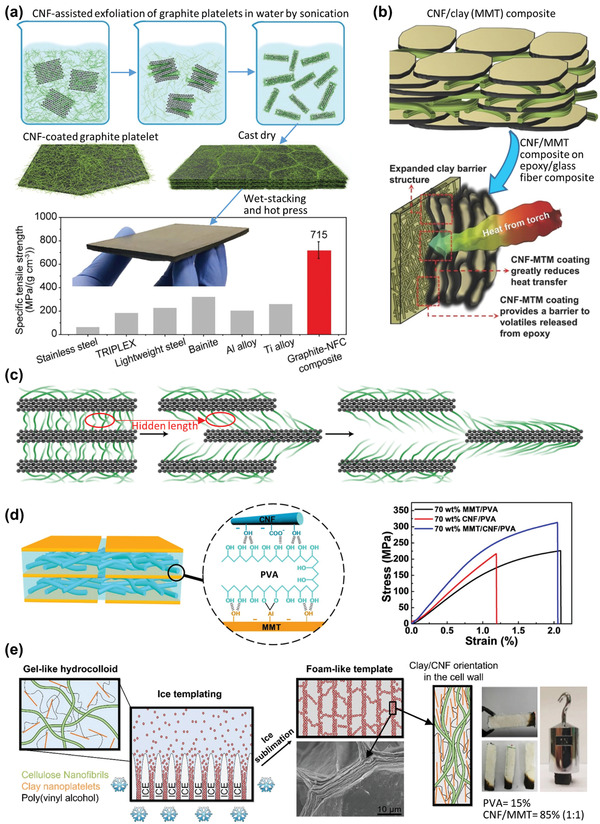
Brick‐and‐mortar facile interfacial engineering for high‐performance nanocellulosic materials. a) A binary CNF/graphite (1:1) thick, light‐weight, strong, and tough nanocomposite from their aqueous blend. Reproduced with permission.^[^ ^207^ ^]^ Copyright 2019, Elsevier. b) A binary CNF/clay (montmorillonite, MMT) nanocomposite from their aqueous blend exhibits excellent fire/thermal shielding property by showing intumescent behavior. Reproduced with permission.^[^ ^194^ ^]^ Copyright 2016, Wiley‐VCH. c) Schematic showing the fracture process of the brick‐and‐mortar‐structured nanocellulose composite (here, as a case example, graphite–CNF composite) under tension. The deformation proceeds through mutual sliding of the nanocellulose‐coated platelets via breaking and reforming of new hydrogen bonds and stretched‐out of the “hidden lengths”. Reproduced with permission.^[^ ^207^ ^]^ Copyright 2019, Elsevier. d) A ternary CNF/MMT/poly(vinyl alcohol) (PVA) nanocomposite from their aqueous blend showing the combined effect of the brick‐and‐mortar structure and an extra lubricating polymer in the composition. Reproduced with permission.^[^ ^208^ ^]^ Copyright 2014, American Chemical Society. e) A CNF/MMT/PVA ternary foam with brick‐and‐mortar‐structured cell‐walls via ice‐templating that shows extraordinary fire retardancy along with high strength (supports weight ever after complete burning in the cone calorimetry tests). Reproduced with permission.^[^ ^202^ ^]^ Copyright 2019, Elsevier.

Recent literature suggests that mimicking brick‐and‐mortar structure is perhaps the most plausible and fast strategy to generate thick, light‐weight, strong, stiff, and tough nanocellulosic composites for potential structural applications. Several strategies have been developed to construct brick‐and‐mortar‐like nanocellulosic materials such as self‐assembly of components in water media followed by dehydration, layer‐by‐layer sequential assembly, or ice‐templating/freeze‐casting/bidirectional freezing (**Figure** **12**).^[^ ^190^, ^192^, ^193^, ^194^, ^195^, ^196^, ^197^, ^198^, ^199^, ^200^, ^201^, ^202^, ^203^, ^204^, ^205^, ^206^, ^207^, ^208^ ^]^ Therein, nanocelluloses were mainly self‐assembled as the mortar between the inorganic nanoplatelets, such as clay, MXene, talc, graphite, graphene, or graphene oxide, in a binary blend system (Figure 12a,b). In an aqueous suspension, nanocelluloses were able to exfoliate platelets, e.g., graphite and graphene (Figure 12a).^[^ ^201^, ^207^ ^]^ Nanocelluloses formed a coating on the platelets mainly through physical interactions, thus, sterically ensured formation of a stable uniform dispersion of the platelets (such as by electrostatic repulsion). Thereupon, the blends were mostly vacuum‐filtered or casted to obtain self‐assembled brick‐and‐mortar‐structured composites. Such a facile processing led to strong and dynamic hydrogen‐bonded nanocellulosic interfaces between the platelets that caused synergistic improvement in strength, stiffness, and toughness of the corresponding nanocomposites. Upon deformation, these nanocellulosic interfaces experience considerable friction owing to the tangled network locked by hydrogen bonds. The deformation proceeds through mutual sliding of the nanocellulose‐coated platelets via breaking and reforming of new hydrogen bonds, i.e., they work as sacrificial bonds (Figure 12c).^[^ ^207^, ^209^ ^]^ Also, one may propose that nanocelluloses, especially the long flexible CNFs, in a tangled network system are not completely stretched‐out, thereby, they progressively become stretched‐out during deformation/elongation, i.e., act as “hidden lengths,” which absorb more energy than breaking bonds (Figure 12c).^[^ ^209^ ^]^ However, in ternary compositions, extra components, such as polymers, diblock proteins (equipped with CBMs and platelet‐interacting functions), or graphene (as a lubricating agent), were used that reduced interfacial friction during mutual sliding of the nanocellulose‐coated platelets, enhanced the bonding interactions between the nanocelluloses and the platelets, or provided hidden lengths.^[^ ^196^, ^198^, ^199^, ^202^, ^203^, ^205^, ^208^ ^]^ Thereby, the interfacial stress transfer ability was maximized (i.e., strengthening and stiffening), and the crack initiation and progress were delayed, deflected, and bridged (i.e., toughening) (Figure 12d).

By mimicking synergistic brick‐and‐mortar structure, excellent mechanical properties of the nanocellulosic composite materials have been achieved. For binary nanocomposites (excluding those contained nanocarbon materials in the composition), the σ of up to ≈200 MPa and *E* of up to ≈20 GPa were reported. These materials are suitable for semi‐structural applications,^[^ ^23^ ^]^ and show excellent fire/thermal shielding (CNF/clay) (Figure 12b),^[^ ^193^, ^194^, ^200^ ^]^ electromagnetic interference shielding (CNF/MXene),^[^ ^192^ ^]^ iridescence (CNC/poly(vinyl alcohol)),^[^ ^190^ ^]^ or barrier properties.^[^ ^197^, ^200^ ^]^ For a ternary CNF/clay/poly(vinyl alcohol) composite, for example, the σ and *E* reached to ≈300 MPa and ≈23 GPa, respectively, with a toughness of ≈4 MJ m^−3^ at 70 wt% CNF/clay (2:1) reinforcement (Figure 12d).^[^ ^208^ ^]^

However, extraordinary mechanical properties were achieved by combining carbon‐based nanomaterials and nanocelluloses.^[^ ^196^, ^201^, ^204^, ^205^, ^207^ ^]^ For example, the synergistic mechanism led to a light‐weight (ρ only 1.2 g cm^−3^) binary nanocomposite of CNF/graphite (1:1) with an incredible σ of >1 GPa and toughness of up to ≈30.0 MJ m^−3^.^[^ ^207^ ^]^ The specific strength of the nanocomposite was 794 MPa g^−1^ cm^3^, which was significantly greater than most engineering materials, e.g., steels, aluminum, and titanium alloys (Figure 12a). Further, several sheets of these nanocomposites could be stacked to form a thick (demonstrated up to 3 mm), high‐strength structural materials (Figure 12a). In another study, the brick‐and‐mortar‐like LBL nanocomposite films of CNCs (37%), reduced graphene oxide (59%) and PEI (4%) achieved an σ of up to ≈750 MPa with an astronomical *E* of up to ≈200 GPa, which is probably the best known *E* value for any man‐made nanocellulosic materials.^[^ ^205^ ^]^ These nanocarbon‐containing materials also show excellent electrical properties intuitively. Besides promising structural materials, ultralight, strong, and tough foams,^[^ ^202^ ^]^ aerogels,^[^ ^199^, ^206^ ^]^ and fibers/filaments^[^ ^203^ ^]^ were also prepared. In particular, foams and aerogels of CNF/clay/poly(vinyl alcohol) are of practical significance in terms of their dual functions, i.e., load bearing and fire retardancy (Figure 12e).^[^ ^202^ ^]^ These foams are ecofriendly, nontoxic and recyclable, and have similar flame retardancy as industrially available phenolic foams.

One may note that the nanocellulosic reinforcement of engineering thermoplastics, such as polyamide, polyvinyl chloride, polyethylene, polystyrene, polypropylene, etc., are of great interest particularly for light‐weight, fuel‐efficient, and hence, low‐carbon‐emitting vehicles.^[^ ^210^ ^]^ Therein, it can be envisaged that by employing the idea of Pickering stabilization technique,^[^ ^166^, ^168^ ^]^ nanocellulose/microplastic brick‐and‐mortar assembly could possibly be made towards strong, stiff, and tough engineering plastics.

### 6.4 Ligno‐Nanocellulosic Materials

**Figure 13:**
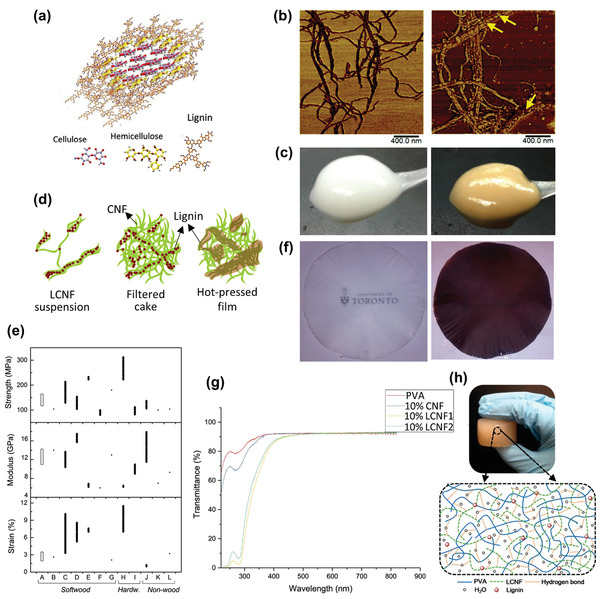
Ligno‐nanocellulosic materials: characteristics and properties. a) Schematic cross‐section of a model cellulose fibril in the native state. Adapted with permission.^[^ ^248^ ^]^ Copyright 2019, Springer Nature. b) AFM image of lignin‐free CNFs (left) and LCNFs (right). The arrows point to some of the lignin nanoparticles, which act as a cementing material between the cellulose nanofibrils. Adapted with permission.^[^ ^226^ ^]^ Copyright 2015, Royal Society of Chemistry. c) Photograph of concentrated lignin‐free CNF suspension (left) and LCNF suspension (right). Adapted with permission.^[^ ^213^ ^]^ Copyright 2020, Springer Nature. d) Schematic of LCNF film formation via hot‐pressing. The lignin melts and forms continuous matrix reinforced by CNFs. Adapted with permission.^[^ ^226^ ^]^ Copyright 2015, Royal Society of Chemistry. e) Mechanical properties of films prepared from LCNF (A) versus films prepared from lignin‐free CNFs. The lignin‐free CNFs were prepared from softwood sulfite pulp (B), softwood dissolving grade fibers (C), softwood bleached sulfite pulp (D), softwood dissolving grade and bleached sulfite fibers (E), softwood bleached Kraft pulp (F), bleached spruce sulfite pulp (G), hardwood bleached Kraft pulp (H); birch Kraft pulp (I), swede root pulp (J), sugar beet pulp (K), and palm fruit bunch fibers (L). Reproduced with permission.^[^ ^226^ ^]^ Copyright 2015, Royal Society of Chemistry. f) Appearance of a lignin free CNF film (left) and a LCNF film (right). Adapted with permission.^[^ ^240^ ^]^ Copyright 2017, Elsevier. g) Light transmittance spectra of PVA composites showing UV‐blocking property. Adapted under the terms of the CC‐BY Creative Commons Attribution 4.0 International license (https://creativecommons.org/license/by/4.0).^[^ ^251^ ^]^ Copyright 2019, The Authors, published by American Chemical Society. h) Photograph of LCNF/PVA hydrogel and the schematic of interactions among the components. Adapted with permission.^[^ ^254^ ^]^ Copyright 2018, American Chemical Society.

As already mentioned, the main raw materials for nanocellulose production are the woody and non‐woody plants, which are principally composed of cellulose (≈40–45%), hemicelluloses (≈20–35%), and lignin (≈20–35%)^[^ ^211^ ^]^ (Note that some non‐woody sources contain high cellulose content, such as cotton, hemp, etc.). The cellulose fibrils are surrounded by hemicelluloses and cemented together by lignin (**Figure** **13**a). Lignin is a complex 3D polymer of aromatic compounds, which is hydrophobic by nature compared to cellulose and hemicelluloses.^[^ ^212^ ^]^ The generally adopted process for nanocellulose production is to fully remove lignin and partially remove hemicelluloses by various chemical pulping and bleaching reactions to access the cellulose fibrils followed by mechanical nanofibrillation or hydrolysis. This way we obtain nanocelluloses with high purity but at the expenses of low yield (40–50% or even lower),^[^ ^213^ ^]^ high environmental hazards, and high production cost. The limitations of these nanocelluloses in material formation are already known and discussed, such as difficulties in dewatering, redispersibility (hornification), and incompatibility with hydrophobic polymers.

Therein, recently, the concept of ligno‐nanocelluloses or simply lignin‐containing nanocelluloses is receiving great interest. Nanocelluloses that contain >1% lignin can broadly be referred to as the ligno‐nanocelluloses.^[^ ^213^ ^]^ We consider retention of the lignin in the nanocelluloses as a nonmodification surface and interface engineering strategy, because 1) lignin is not a foreign substance, and 2) it can change the surface properties of nanocelluloses, and hence can affect the fibrillation of nanocelluloses and interfacial interactions in the nanocellulosic materials.^[^ ^213^, ^214^ ^]^ Ligno‐CNFs (LCNFs) have been produced directly from fine plant‐based particles (such as wood flour, bamboo particles, etc.) and mechanical pulps via mechanical grinding, homogenizing, or milling/refining with yield as high as 97%.^[^ ^215^, ^216^ ^]^ However, the degree of fibrillation is reported to be lower compared to that of the lignin‐free CNFs. Because, the native hydrophobic lignin surrounding the cellulose fibrils lock them together and prevent swelling, therefore, hinder fibrillation.^[^ ^213^, ^215^, ^217^, ^218^ ^]^ A similar trend can also be observed for the semichemical pulps.^[^ ^219^, ^220^ ^]^ Although, thin LCNFs (≈3–10 nm in diameter) can be obtained by introducing charge groups by esterification or TEMPO‐oxidation in the starting lignocellulosic source before mechanical fibrillation.^[^ ^219^, ^221^, ^222^ ^]^ However, fine ligno‐CNCs (LCNCs) can be directly obtained from the lignocellulosic samples or thermomechanical pulps by hydrolysis with the acidic deep eutectic solvent or H_2_SO_4_ with a high lignin content (as high as ≈50%) and yield (as high as ≈70%).^[^ ^223^, ^224^ ^]^ On the other hand, interestingly, the residual lignin in unbleached chemical pulps (such as Kraft pulp, alkaline‐treated pulp, etc.) facilitates mechanical nanofibrillation of LCNFs below 15 nm with a trend of decreasing LCNFs diameter with increasing residual lignin content.^[^ ^225^, ^226^, ^227^ ^]^ For example, the LCNFs with 14% residual lignin achieved a diameter of ≈5 nm compared to a diameter of >10 nm for LCNFs with 7% residual lignin.^[^ ^225^ ^]^ The residual lignin present in chemical pulps is significantly degraded and modified (probably less hydrophobic than native lignin) and present in much lower quantities as precipitated nanoparticles (Figure 13b), therefore facilities fiber swelling and fibrillation.^[^ ^218^, ^225^, ^226^, ^227^, ^228^ ^]^ Also, hemicelluloses are present in higher quantity in the unbleached chemical pulps, which are known to facilitate nanofibrillation.^[^ ^19^, ^229^ ^]^ Another proposal is that the residual lignin can act as an antioxidant that scavenges reactive cellulose free radicals to counteract recombination of cellulose fragments, hence promotes nanofibrillation.^[^ ^213^, ^225^, ^227^ ^]^ Ligno‐nanocelluloses are brownish in color that inherits from the lignin itself (Figure 13c), and have crystallinity lower than that of lignin‐free nanocelluloses because of having high amount of non‐cellulosic constituents. The viscosity of LCNFs was reported lower compared to that of lignin‐free CNFs.^[^ ^220^, ^222^ ^]^

The most extensively studied material of ligno‐nanocelluloses is their films. Lignin is known as the thermoplastic material that softens upon heating at ≈135 °C at 5% moisture content, and at 80–90 °C at 20–40% moisture content.^[^ ^213^, ^230^ ^]^ Therefore, the lignin in LCNF films melts during hot‐pressing and forms a continuous matrix reinforced by the CNFs (Figure 13d). One might expect lignin to reduce fiber–fiber bonding. But, well‐distributed lignin works as an agent of stress transfer so mechanical properties are not degraded.^[^ ^226^ ^]^ On the other hand, it increases hydrophobicity and decreases oxygen permeability of the films.^[^ ^226^ ^]^ The mechanical properties of LCNF films are comparable to those of lignin‐free CNF films (Figure 13e).^[^ ^220^, ^226^ ^]^ A high lignin content (e.g., 21 wt%) in the LCNF film improves its thermal stability and wet strength.^[^ ^231^ ^]^ Other studies also described the LCNF films having properties of high mechanical strength,^[^ ^219^, ^232^, ^233^, ^234^, ^235^, ^236^, ^237^, ^238^, ^239^ ^]^ wet strength,^[^ ^232^, ^235^ ^]^ hydrophilicity (high water contact angle),^[^ ^219^, ^232^, ^233^, ^234^, ^236^, ^237^ ^]^ water resistance,^[^ ^232^, ^235^, ^237^, ^239^ ^]^ dewatering speed,^[^ ^232^ ^]^ thermal stability,^[^ ^232^, ^234^, ^235^, ^236^, ^239^ ^]^ and redispersibility.^[^ ^236^, ^238^ ^]^ The LCNF films usually appear dark brown in color (e.g., at 23–28 wt% lignin content)^[^ ^220^, ^240^ ^]^ with no optical transparency due to the light absorption and scattering by the lignin (Figure 13f).^[^ ^241^ ^]^ However, recently, due to the successful preparation of LCNFs with a diameter <5 nm, a brownish semi‐transparent LCNF film has been demonstrated with a total light transmittance of 82% at 650 nm wavelength at a lignin content of ≈14 wt%.^[^ ^225^ ^]^

To produce LCNF composites is a new trend of nanocellulose‐based materials owing to the interesting features of LCNFs. The LCNFs and LCNCs, both, have been investigated as the reinforcing filler for the poly(lactic acid) (PLA).^[^ ^224^, ^242^, ^243^ ^]^ The presence of hydrophilicity in ligno‐nanocelluloses imparted a strong compatibility with the PLA matrix and hence an improved dispersion. Therefore, not only the mechanical and dynamic thermomechanical (storage modulus) properties of the PLA were improved, but also surface polarity was reduced and 3D‐printability was improved. For instance, the addition of 10 wt% LCNFs in the PLA matrix increased the modulus and strength by 88% and 111%, respectively, and the water vapor transmission rate was reduced by 52%.^[^ ^243^ ^]^ Similarly, LCNF‐reinforced PCL and PS show a simultaneous increase in stiffness, strength, and toughness in contrast with lignin‐free CNF reinforced composites.^[^ ^244^, ^245^ ^]^ Advantageously, even the dried LCNFs power can be compounded with PCL, which results in a more favorable composite tensile performance.^[^ ^245^ ^]^ This is not possible with the lignin‐free CNFs due to their hornification in dried state. It was reported that the hydroxyl groups of LCNFs could react with epoxy, polymeric diphenylmethane diisocyanate (PDMI), and fluoroalkyl silane.^[^ ^240^, ^246^, ^247^ ^]^ The obtained LCNFs/PDMI system showed an enhanced mechanical performance as a wood adhesive.^[^ ^246^ ^]^ The improvement of mechanical properties after incorporation of LCNF into poly (butylene adipate‐co‐terephthalate) and PP have also been observed.^[^ ^215^, ^248^, ^249^ ^]^ In terms of advanced functional properties, LCNFs bestowed antioxidant characteristic, highly optical transparency, strong UV‐absorbing capability to the PVA matrix composite.^[^ ^250^, ^251^ ^]^ In particular, at only 10 wt% LCNFs, the PVA composite transmitted ≈50% (on average), <10% and 2–6% light in the UV‐A (315−400 nm), UV‐B (280−315 nm), and UV‐C (200–280 nm) regions, respectively (Figure 13g). In contrast, the pure PVA and lignin‐free‐CNFs/PVA transmitted 79% and 68% light, respectively, even at the wavelength of 280 nm (UV‐C). This result suggests that the LCNFs, apart from enhancing the mechanical performance of polymers, functions as a UV‐absorbing component, which could find potential applications in optical parts such as lenses and optoelectronics.

LCNFs have been used to form reinforced PVA hydrogel (Figure 13h) with a 17‐fold increase in storage modulus and a fourfold increase in specific Young's modulus at only 2 wt% loading.^[^ ^252^ ^]^ It is suggested that the lignin can reduce depolymerization of cellulose fibrils during LCNF production, resulting in high aspect ratio of LCNFs, which can promote physical bridging and entanglement in the hydrogel network. Further, the properties of the hydrogel can be tuned simply by varying the LCNF content or the lignin content in the LCNFs. In a different study, LCNFs were used to stabilize air–liquid interfaces to form strong liquid foam with a stability five times higher than that of surfactant‐only foam. The LCNF showed good interaction with the surfactant that created an elastic interface that gave the foams superior ability to transport in porous media without the plugging issue. Therefore, these foams are excellent candidates for robust oil recovery processes.^[^ ^253^ ^]^

Finally, the ligno‐nanocelluloses consume less chemical and energy to prepare, cause less environmental impact, naturally possess characteristics similar to hydrophobically modified nanocelluloses, and are low cost. The potential and advantages of ligno‐nanocelluloses in formation of nanocellulosic materials have already been identified as discussed above. Therefore, they have a high possibility to take over the role of conventional nanocelluloses in certain materials such as structural and semi‐structural composites, packaging materials, and so on.

## 7 Conclusions

In this report, we reviewed recent progress in surface and interface engineering of nanocelluloses from the novel aspects, in which we elaborated on the nanocellulose‐, chemistry‐ and process‐oriented engineering strategies. We not only covered chemical surface engineering of nanocelluloses, but also more environmentally benign intelligent approaches for the fabrication of nanocellulosic advanced materials.

In terms of chemical modification, the characteristic surface chemistries and dispersibility of nanocelluloses are of great importance in order to select an appropriate reaction route. A new insight into the stage‐oriented chemical modification of nanocelluloses such as before and after nanocellulose isolation or during material and after material formation has been discussed to widely open the opportunities for the future engineering of nanocelluloses for a wide range of applications, including structural and semistructural composites, hydrogels, foam, 3D printing ink, films and filaments. We note that the identification of the best route to form a nanocellulosic material is critical because it may not only affect the material properties but also the time, costs, resources, and environment. For example, extracted nanocelluloses can be modified before material formation; in contrast, simultaneous nanofibrillation, modification and material formation route would take much less resource, time, and cost, and would have high industrial scalability.

When it comes to the practical application, the nature is not a best friend for nanocelluloses. It is their inherent hydrophilicity that is to be tackled. Hydrophobic surface modification has widely been accepted as the best route. The reaction medium for this modification is almost always based on organic solvents that increases the cost and human hazards let alone the environmental concerns. Also, it is expected that being hydrophilic in nature, nanocelluloses probably do not prefer nonpolar medium. They remain inhomogeneously distributed and tend to agglomerate in nonpolar medium that may cause ineffective and inhomogeneous surface modification. In this aspect, we particularly admire the hydrophobic modification of nanocelluloses in aqueous media as well as the enhanced modification approaches, e.g., modification in the solvent‐free condition, which we have discussed with great importance.

Meanwhile, nonmodification interface engineering, such as impregnation, Pickering emulsion and brick‐and‐mortar approaches, provides effective ecofriendly solutions for processing of high‐performance high‐volume nanocellulosic materials for structural and semistructural applications. Best known nanocellulosic materials in terms of mechanical properties have been achieved by mimicking brick‐and‐mortar structure. On the other hand, Pickering emulsion approach and impregnation method provide facile opportunity for construction of functional nanocellulosic materials from immiscible components. As for imparting hydrophobicity to nanocelluloses, a simple but powerful concept is retaining natural lignin on to the nanocellulose surface. The lignin‐containing nanocelluloses are benefited from high yield combined with lower production cost and environmental hazard by reducing chemical consumption. Consequently, chemical surface modification of nanocelluloses should not be overwhelmingly considered a one‐way route for forming good interfaces between nanocelluloses and (immiscible) matrices, and for imparting functionalities towards high‐performance and functional nanocellulosic materials.

It may be frustrating that nanocomposites have not been widely commercialized, no matter they are made from nanocellulose, carbon nanotube or nanoclay. However, we predict that nanocellulose‐based products will become much more available in our everyday life in the next decade considering the recent enormous attention paid in the nanocellulose R&D. We hope that this article may inspire readers to come up with new concepts on processing of the nanocellulosic advanced materials.

## Conflict of Interest

The authors declare no conflict of interest.
